# A machine learning model for predicting ischemic and bleeding risk after percutaneous coronary intervention: Development and external validation

**DOI:** 10.1371/journal.pone.0353066

**Published:** 2026-09-15

**Authors:** Hassa Iftikhar

**Affiliations:** Department of Internal Medicine (Cardiology), Tongji Medical College, Huazhong University of Science and Technology, Hubei, Wuhan, China; Azienda Ospedaliero Universitaria Careggi, ITALY

## Abstract

**Objective:**

This study aimed to develop and externally validate a machine learning-based risk prediction model of ischemia and bleeding events in patients receiving percutaneous coronary intervention (PCI) and dual antiplatelet therapy (DAPT) and, to evaluate its clinical potential and economic implications compared with existing risk scoring systems.

**Methods:**

A weighted LightGBM model was trained on a PCI cohort from the United Arab Emirates, comprising 4,812 participants, and then externally validated on the MIMICIV database (3,406 patients). The main outcomes were composite ischemic events and major bleeding events. The model discrimination, calibration, and clinical utility were evaluated using calibration plot, AUROC, and decision curve analysis. This model was used to estimate economic results under hypothetical risk-guided management strategies.

**Results:**

The weighted LightGBM model achieved AUROC values of 0.87 for ischemic events and 0.85 for bleeding events during internal validation. In external validation, AUROC values were 0.84 and 0.82, respectively. These results were higher than discrimination performance of the established scoring systems, including DAPT and PRECISE-DAPT. Exploratory analyses suggested that risk-guided strategies informed by the model may have potential, although these findings require prospective validation.

**Conclusions:**

The explainable AI model demonstrated good discrimination and calibration for post-PCI ischemic and bleeding risk prediction. The model showed higher predictive performance compared with conventional risk scores and may serve as a potential decision-support tool to inform future prospective validation evaluating its clinical utility.

## Introduction

Acute coronary syndrome (ACS) remains a major global health burden and is particularly crucial in the UAE, where cardiovascular disease (CVD) contributes to morbidity and mortality rates. Following coronary intervention (PCI), the selection of dual antiplatelet therapy (DAPT) duration require balance to minimize ischemic risk and manage, the risk of bleeding. [1] Innovation in drug-eluting stents (DES) and risk scoring based on personalized risk factors have assisted in clotting prevention, but the ability to balance clotting prevention and minimize bleeding still remains complicated. [2,3] This problem is particularly relevant in the UAE where a diverse population are highly heterogenous by genetic, metabolic, and lifestyle variations. [4] Although the period of DAPT should be individualized due to competing risks, there is no current practice of individualization, and the widely used protocols or fixed risk scores are PRECISE-DAPT and DAPT. These models are limited by their inability to dynamically adapt to evolve patient profiles and their multifaceted interactions among risk factors. [5] This research gap highlight the need for improved risk stratification approaches capable of integrating multidimensional medical information to support personalized DAPT decision-making beyond one-size-fits-all paradigms. [6]

Although widely used, PRECISE-DAPT and DAPT scores offer, limited predictive accuracy for individual patients. Machine learning methods capture complex nonlinear relationships among clinical variables and has the potential to improve risk prediction beyond conventional risk scores. [7] Similar observations have been reported in several East Asian population, where patients often emphasize the necessity of flexible treatment, demonstrate lower thrombotic risk but are highly bleeding susceptible during the antiplatelet therapy. [8] Therefore, fixed-duration DAPT regimens may be poorly suited to such heterogeneous populations, highlighting the importance of enhanced individualized risk stratification. [9] Risk prediction models based on machine learning can incorporate diverse patient data to estimate stent restenosis and other morbidities with higher accuracy. [10] This study focuses on clinical, procedural, and laboratory predictors rather than imaging-based or multi-omics inputs, which remain an important area for future investigation. Such approaches may be particularly useful in regions such as the UAE and Oman, where different populations and genetic conditions require a more delicate approach to DAPT strategies to improve the risk stratification and support adherence to guideline-based therapy decisions. [11]

Recent machine learning models have shown improved discrimination for predicting ischemic and bleeding events after PCI compared with traditional risk scores. [12] These models offer the potential to support dynamic post-PCI risk stratification by accounting for evolving comorbidities and treatment-related factors. [13] In addition, the potential clinical and economic implications of risk-guided strategies remain insufficiently explored. [14,15] This evidence gap is particularly relevant in the Arabian Gulf, where DAPT strategies derived from guidelines may not fully reflect population-specific risk profiles, contributing to variability in clinical decision-making and outcomes. Therefore, multicohort validation across distinct healthcare systems, combined with economic evaluation is essential to assess the generalizability and practical relevance of risk-guided DAPT personalization. [16]

Existing AI models for cardiac event prediction, such as those developed for the Malaysian population, demonstrate the capability of machine learning frameworks to create generalizable risk assessments for guiding revascularization strategies and improving patient safety. [17] Consequently, the development of robust AI models for DAPT duration personalization necessitates multicenter data from diverse cohorts to ensure broad applicability and equitable performance across different ethnic and genetic backgrounds prevalent in the UAE. [18] The economic burden of CVD adverse events cases in the United Arab Emirates, further supports the need of cost-efficient and tailored dual antiplatelet therapy (DAPT) interventions that could inform clinical-decision making. [19] Although prior studies have demonstrated higher discrimination performance of AI models for PCI-related outcomes, including bleeding risk, restenosis, and mortality, downstream clinical and economic implications remain insufficiently characterized. Genetic variability affecting platelet reactivity and antiplatelet metabolism adds further complexity, reinforcing the need for cautious, exploratory evaluation of AI-driven strategies and continued validation across diverse populations. Moreover, the predictive properties of traditional models based on nonlinear relations and higher-order interactions between various risk factors can be difficult to capture, thus restricting their personal-level predictive validity and epidemiological significance. Moreover, changes in platelet reactivity and the dynamic nature of the current knowledge regarding the relationship between genetics and the effect of DAPT require a constant revision of models and validation of this relationship using prospective and real-life data in different populations. [20–22]

Nevertheless, the integration of AI models in the clinical setting of cardiovascular care continues to be complicated by several issues such as ethics, model interpretability, and cost-effectiveness of the mass application. [23] These considerations are particularly relevant in heterogeneous healthcare environments, such as the UAE and neighboring Gulf countries, where robust external validation and careful evaluation of clinical utility are required due to population diversity and practice variability. [24] Accordingly, this study aimed to develop and externally validate a machine learning-based prediction model for ischemic and major bleeding events following PCI using routinely available clinical, procedural, and laboratory variables. The model was developed using a UAE PCI cohort and externally validated using the MIMIC-IV database. Model performance was compared with established risk scores, and exploratory analyses were conducted to evaluate potential differences in risk-guided management strategies using simulation-based projections. This work is intended as a proof-of-concept evaluation to inform future prospective studies, rather than to provide recommendations for practice-changing recommendations.

## Method

### Study design and data sources

This study employed a retrospective, multi-cohort analytical design to develop and externally validate a machine learning-based prediction model for post-percutaneous coronary intervention (PCI) ischemic and bleeding risk stratification. A prespecified development-validation framework was used to minimize overfitting and evaluate the transportability of the model. Two independent data sources were used to achieve the desired methodological rigor and external validity. The development cohort comprised a secondary, de-identified UAE clinical dataset obtained under institutional data governance arrangements for research purposes. The authors accessed the dataset in anonymized form and were not the data custodians. The dataset included structured electronic health record (EHR) information on demographics, comorbidities, laboratory parameters, procedural characteristics, and post-PCI outcomes. The Medical Information Mart for Intensive Care IV (MIMIC-IV) database, a publicly available, de-identified critical-care dataset of longitudinal clinical data of a large tertiary healthcare system, was used for external validation to evaluate model generalizability across distinct clinical environments and case settings. The use of independent development and external validation cohorts was prespecified to assess robustness and transportability across heterogeneous healthcare systems. This study represents a retrospective, multicohort validation of a machine learning prediction model and is intended to be hypothesis-generating rather than intervention.

### Ethics statement

This study was conducted using retrospective, fully de-identified datasets and did not involve direct patient contact. Access to the MIMIC-IV database was obtained through the PhysioNet Credentialed Health Data Access process after completion of the required training and certification. Clinical data from the UAE cohort were accessed for research purposes between January 2023 and May 2024. No direct patient identifiers were accessible to the investigators during or after data collection, and no attempts were made to re-identify individual participants.

### Study population and outcome

Sample size was determined by the availability of eligible patients within the UAE development cohort and MIMIC-IV validation cohort. The number of outcome events exceeded commonly recommended minimum events-per-predictor thresholds for machine learning-based prediction model development, supporting model stability and reducing risk of overfitting. Adult patients who underwent PCI and were later exposed to dual antiplatelet therapy were included. Patients with incomplete follow-up data, incomplete key outcome variables, and incomplete clinical documentation were excluded. The primary outcomes were as follows:

1. Ischemic events are a combination of myocardial infarction, stent thrombosis, ischemic stroke, or cardiovascular death.
2. Bleeding events, delineated by bleeding academic research consortium (BARC) type 3 or type 5, agree with DAPT outcome study and guideline-supported definitions. [25]

Identical outcome definitions were applied across both the UAE development cohort and the MIMIC-IV external validation cohort using harmonized diagnostic and clinical coding mappings. The Follow-up duration extended from the index PCI to the occurrence of an outcome event or censoring at the end of the available follow-up period. The analytic cohorts had a median follow-up period of approximately 37 months, which allowed the determination of both immediate and long-term post-PCI risk. Clinical outcomes were identified using diagnostic codes, procedures documentation, and hospitalization records. For MIMIC-IV dataset, phenotyping algorithms were constructed using ICD-9 and ICD-10 code mappings aligned with published cardiovascular outcome definitions. ICD-9 and ICD-10 based phenotyping algorithms were identically applied in both cohorts using harmonized coding mappings. The Detailed code lists were used to identify stroke, myocardial infarction, stent thrombosis, and BARC-defined bleeding events are provided in S1 Table. Sensitivity analyses using alternative outcome definitions were conducted to evaluate robustness to potential coding variability.

### Feature selection and data preprocessing

The candidate predictors are listed in S2 Table and included demographic, clinical, procedural, laboratory, and treatment variables. No post-hoc outcome-driven variable selection was performed to avoid information leakage and preserve the integrity of external validation. Before constructing the model, the data preprocessing steps involved the normalization of continuous variables, and one-hot encoding of categorical variables. Missing data were handled using multiple imputation by chained equations (MICE) (S2 Table). The proportion of missing imputation for each variable was assessed. Variables with >30% missing imputation were excluded from modeling. Total 10 imputed datasets were generated and combined using Rubin’s rules:

- Continuous variables with minimal missing (i.e., body mass index) were imputed using median values.
- Binary comorbidities were not explicitly documented and assumed absent, consistent with prior electronic health record-based prediction studies.

Feature inclusion was restricted to variables available at or shortly after PCI to preserve real-world clinical applicability. Genetic variables were accessible to the subsets of the development cohort, were considered exploratory characteristics, and were not required for model deployment. The imbalance between classes was balanced through training outcome-specific weighting, thereby preventing resampling and information leakage. All preprocessing steps were performed using reproducible pipelines, to ensure identical transformations across validation cohort. Consistent outcomes were obtained from sensitivity analyses including imputation strategies, complete-case modeling, and exclusion of genetic variables (S2 Table).

### Model development and validation

A supervised machine learning approach was applied to predict ischemic and bleeding risk across prespecified post-PCI time windows (0–3, 3–6, 6–12, and 12–36 months)**.** The weighted gradient-boosting decision tree algorithm (LightGBM) was selected as the primary predictive model for its ability to handle nonlinear interactions and heterogeneous feature types while maintaining interpretability. Model training was performed using stratified k-fold cross-validation (k = 5) in the UAE development cohort to optimize hyperparameters and assess internal performance. Internal validation metrics were derived from cross-validation folds to estimate model stability. Hyperparameter tuning for the LightGBM model was conducted using five-fold cross-validation within the training dataset. A randomized strategy was used to optimize key parameters including number of estimators, learning rate, maximum tree depth, and feature fraction. The model selection was evaluated based on maximizing cross-validated AUROC. The trained model was applied without recalibration to the independent MIMIC-IV cohort to evaluate transportability across heterogeneous clinical populations. Differences in baseline characteristics between the development and validation cohorts were examined descriptively to assess potential distributional shifts that could influence model performance. Model calibration was evaluated using calibration intercept, calibration slope, and visual calibration plots, with bootstrap resampling applied to estimate uncertainty. Logistic regression was developed as a benchmark linear model using the same predictor set as the machine learning models to ensure fair comparison. Additional comparator models included random forest and neural network architectures. These comparator models were developed exclusively for performance benchmarking and were not used in downstream clinical or economic simulations. External validation in the independent, geographically and clinically distinct MIMIC-IV cohort was conducted in accordance with recommended best practices for retrospective AI prediction model evaluation.

### Model explainability and clinical interpretability

To enhance transparency and clinical interpretability, explainable AI techniques were applied to the primary model. Feature contribution was assessed using SHapley Additive exPlanations (SHAP), enabling both global and individual-level interpretation of model predictions. The global SHAP feature importance rankings were used to quantify the relative contribution of individual predictors to ischemic and bleeding risk predictions (S6 Fig). These analyses were used to confirm clinical plausibility and identify key risk drivers. SHAP analyses were prespecified and limited to the main effects in the primary output, with extended dependence analyses reported in supplementary materials.

### Clinical utility assessment

The clinical usefulness of AI-based predictions was evaluated using time-to-event and decision curve analyses. Kaplan-Meier methods were used to compare event-free survival across risk strata. To evaluate a hypothetical AI-guided DAPT strategy, we used predicted ischemic and bleeding risks to simulate hypothetical DAPT duration strategies relative to guideline-based fixed-duration strategies. Specifically, patients with high predicted ischemic risk and low bleeding risk were assigned simulated prolonged DAPT, whereas those with low ischemic risk or high bleeding risk were assigned shorter DAPT durations, with thresholds prespecified from internal validation. Model-based projections of event rates under AI-guided versus guideline-based strategies were derived without altering observed outcomes. Decision curve analysis measured the net clinical benefit of AI-guided risk stratification relative to standard risk scores and treat-none or treat-all strategies across a range of clinically relevant thresholds. All analyses were performed within a time-to-event framework to appropriately interpret censoring and variable follow-up durations. The clinical outcomes represent scenario-based simulations derived from predicted risk distributions rather than observed treatment effects. Therefore, no causal inference framework was applied as these projections should be interpreted as exploratory modeling outputs rather than to be used for therapeutic benefit.

### Economic evaluation

A trial-based health economic evaluation was conducted to explore the potential cost and outcome implications under hypothetical AI-guided DAPT personalization scenarios compared with guideline-based fixed-duration strategies. A simplified decision-analytic framework was used to explore potential costs and health outcomes between AI-guided and guideline-based DAPT strategies. Analyses were performed from a healthcare payer perspective over a time horizon corresponding to the median follow-up duration (approximately 37 months). Costs included antiplatelet therapy, management of major bleeding events, and hospitalization for ischemic event-related events (Table 6). Quality-adjusted life-years (QALYs) were estimated using outcome-specific utility weights derived from previous literature. The incremental cost-effectiveness ratios (ICERs) were calculated as exploratory modeling estimates instead of definitive cost effectiveness results. Both deterministic one-way and probabilistic sensitivity analyses using Monte Carlo simulation were performed to assess the robustness to uncertainty in the key parameters shown in Supplementary S4 and S7 Tables.

### Statistical analysis

Continuous variables were measured as standard deviations and mean or medians with interquartile ranges, whereas categorical variables were reported as percentages and counts. P-values were not used for inferential comparisons because the analyses were primarily predictive. Model performance estimates were reported with 95% confidence intervals where applicable. Analyses were conducted using Python (scikit-learn, LightGBM, and SHAP), with full version details provided in the Supplementary Appendix. Reporting adhered to TRIPOD-AI and contemporary recommendations for machine-learning-based prediction model studies.

## Result

### Study population and cohort derivation

**Fig 1:**
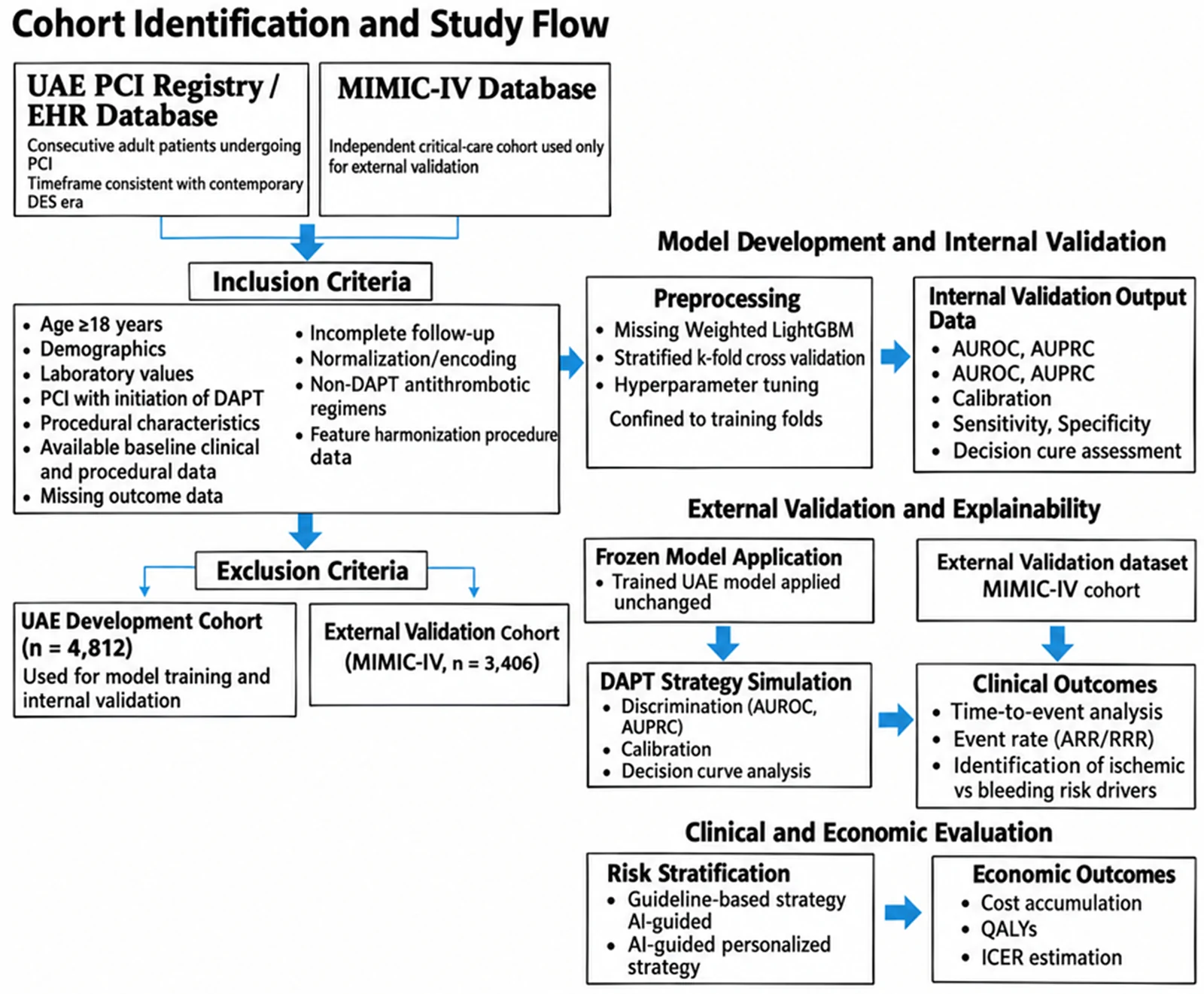
Study design and analytical workflow.

A multicenter UAE PCI registry and a data set of a MIMIC-IV critical care were used as independent data sources to identify a cohort of consecutive patients receiving PCI and dual antiplatelet therapy (DAPT). The final analytic cohorts based on the use of prespecified inclusion, exclusion criteria and the imputation of missing data included a UAE development cohort to train and internally validate the models and an external validation cohort based on MIMIC-IV. Fig 1 shows the cohort derivation process, including exclusions related to the missing key covariates and insufficient follow-up. S5 Fig provides further details and Table 1 summarizes the baseline demographic, clinical, and procedural data of both cohorts. Additional baseline characteristics stratified according to ischemic and bleeding event status are presented in Supplementary S8 Table. The UAE development cohort and external validation cohort differed in several baseline characteristics representing anticipated heterogeneity across health care systems and populations. Baseline differences included age distribution, prevalence of cardiovascular comorbidities, severity of illness, and event rates, reflecting the ICU-enriched nature of the MIMIC-IV cohort relative to the UAE PCI registry (Table 1). Despite these differences, both cohorts included various ischemic and bleeding risk profiles, thereby contributing to the evaluation of model transportability and generalizability.

**Table 1:** Baseline demographic, clinical, and procedural characteristics of patients undergoing percutaneous coronary intervention (PCI) in the UAE development cohort and the external validation cohort derived from the MIMIC-IV database.

Characteristic	UAE Development Cohort (n = 4,812)	External Validation Cohort (MIMIC-IV) (n = 3,406)	P-value†
**Age, years** (mean ± SD)	58.9 ± 10.7	63.4 ± 12.1	<0.001
**Male sex, n (%)**	3,612 (75.1)	2,327 (68.3)	<0.001
**Hypertension, n (%)**	2,986 (62.0)	2,245 (65.9)	0.002
**Diabetes mellitus, n (%)**	2,041 (42.4)	1,097 (32.2)	<0.001
**Atrial fibrillation, n (%)**	384 (8.0)	612 (18.0)	<0.001
**Chronic kidney disease, n (%)**	611 (12.7)	587 (17.2)	<0.001
**Prior bleeding event, n (%)**	298 (6.2)	279 (8.2)	<0.001
**Prior myocardial infarction, n (%)**	1,084 (22.5)	842 (24.7)	0.04
**Multivessel coronary disease, n (%)**	2,176 (45.2)	1,429 (42.0)	0.01
**Complex PCI‡, n (%)**	1,267 (26.3)	824 (24.2)	0.06
**Concomitant oral anticoagulant use, n (%)**	463 (9.6)	718 (21.1)	<0.001
**Baseline planned DAPT duration, months (median [IQR])**	12 [6–2]	12 [6–18]	0.03

† P-values reflect comparisons between cohorts and are provided for descriptive purposes only; no causal inference is implied.

‡ Complex PCI defined as ≥3 stents implanted, ≥ 3 lesions treated, bifurcation PCI with ≥2 stents, chronic total occlusion intervention, or total stent length >60 mm.

*Abbreviations:* PCI, percutaneous coronary intervention; DAPT, dual antiplatelet therapy; CKD, chronic kidney disease; IQR, interquartile range.

### Clinical outcomes and event rates

Prespecified ischemic and bleeding outcomes were consistently defined and applied across both cohorts. Composite ischemic outcomes included myocardial infarction, ischemic stroke, stent thrombosis, and cardiovascular death. Major bleeding outcomes were defined according to Bleeding Academic Research Consortium (BARC) type 3 or type 5 criteria were harmonized across development and validation cohorts using standardized outcome definitions. Table 2 presents the observed cumulative event rates individual and composite ischemic and bleeding endpoints. As expected, ischemic and bleeding events occurred at clinically meaningful frequencies in both cohorts, with moderately higher event rates observed in the external validation cohort, consistent with its critical care-enriched population. These observed outcome distributions provide an appropriate empirical basis for model development, internal validation, and independent external evaluation.

**Table 2:** Clinical outcome definitions and observed event rates for ischemic and bleeding endpoints in the UAE development cohort and the external validation cohort. Composite outcomes were prespecified and applied uniformly across datasets to ensure consistency in model development and validation.

Outcome Category	Outcome	UAE Development Cohort (n = 4,812)	External Validation Cohort (MIMIC-IV) (n = 3,406)
**Ischemic Events**	Composite of myocardial infarction, definite or probable stent thrombosis, ischemic stroke, or cardiovascular death	512 (10.6%)	421 (12.4%)
	Stent thrombosis	74 (1.5%)	63 (1.8%)
	Ischemic stroke	96 (2.0%)	88 (2.6%)
	Myocardial infarction	298 (6.2%)	247 (7.3%)
	Cardiovascular death	144 (3.0%)	169 (5.0%)
**Bleeding Events**	Major bleeding defined according to standardized criteria*	347 (7.2%)	389 (11.4%)
	Intracranial hemorrhage	42 (0.9%)	51 (1.5%)
	Gastrointestinal bleeding	183 (3.8%)	214 (6.3%)
	Other major bleeding	122 (2.5%)	124 (3.6%)
**Follow-up duration**	Median follow-up, months (IQR)	37 (24–48)	36 (18–42)

* Major bleeding was defined using standardized criteria consistent with prior DAPT and PCI studies, including bleeding requiring hospitalization, transfusion, surgical intervention, or associated with hemodynamic compromise.

Percentages reflect cumulative incidence during follow-up and are not mutually exclusive across individual outcome components within each cohort.

Abbreviations: IQR, interquartile range.

### Predictive performance of the primary artificial intelligence model

Table 3 summarizes the discrimination and classification performance of the primary weighted LightGBM model for ischemic and bleeding risk prediction. Model training was conducted exclusively within the UAE development cohort, with cross-validation for internal validation. External validation was performed without recalibration in the independent MIMIC-IV cohort. LightGBM model demonstrated an AUROC of 0.87 (95 percent confidence interval 0.840.89) in ischemic events and 0.85 (95% CI 0.82–0.88) for bleeding events in internal validation. External validation demonstrated AUROC values of 0.84 (95% CI 0.82–0.86) for ischemic outcomes and 0.82 (95% CI 0.79–0.85) for bleeding outcomes. The AI model achieved AUROC values of 0.87 and 0.85 for ischemic and bleeding outcomes in internal validation respectively. The AUPRC values and AUROC area are consistent across cohorts, with expected decreased attenuation during external validation. Specificity, sensitivity, and F1-scores demonstrated equitable classification performance at prespecified thresholds. The time-window-specific performance metrics measured consistent discrimination across early, mid, and late post-PCI periods, thereby supporting the AI-based approach’s temporal robustness (S5 Table).

**Table 3:** Predictive performance of the primary AI model across outcomes and validation cohorts.

Outcome	Dataset	AUROC (95% CI)	AUPRC	Specificity	Sensitivity	F1-Score
**Ischemic Events**	Internal validation (UAE)	0.87 (0.85-0.89)	0.42	0.81	0.79	0.61
	External validation (MIMIC-IV)	0.84 (0.82-0.86)	0.39	0.78	0.76	0.58
**Bleeding Events**	Internal validation (UAE)	0.85 (0.83-0.87)	0.36	0.80	0.77	0.55
	External validation (MIMIC-IV)	0.82 (0.80-0.84)	0.34	0.77	0.74	0.52

Discrimination and classification performance of the primary weighted LightGBM model for prediction of ischemic and bleeding events following percutaneous coronary intervention. Model training was performed exclusively in the UAE development cohort, with internal validation conducted using cross-validation and external validation performed without recalibration in the independent MIMIC-IV cohort.

AUROC values are reported with 95% confidence intervals derived from bootstrapped resampling.

The area under the precision-recall curve (AUPRC) is reported to account for outcome imbalance.

Sensitivity, specificity, and F1-score were calculated using prespecified probability thresholds selected during internal validation and held constant for external validation.

Abbreviations: AUROC, area under the receiver operating characteristic curve; AUPRC, area under the precision-recall curve.

### Comparison with the conventional DAPT risk scores

**Fig 2:**
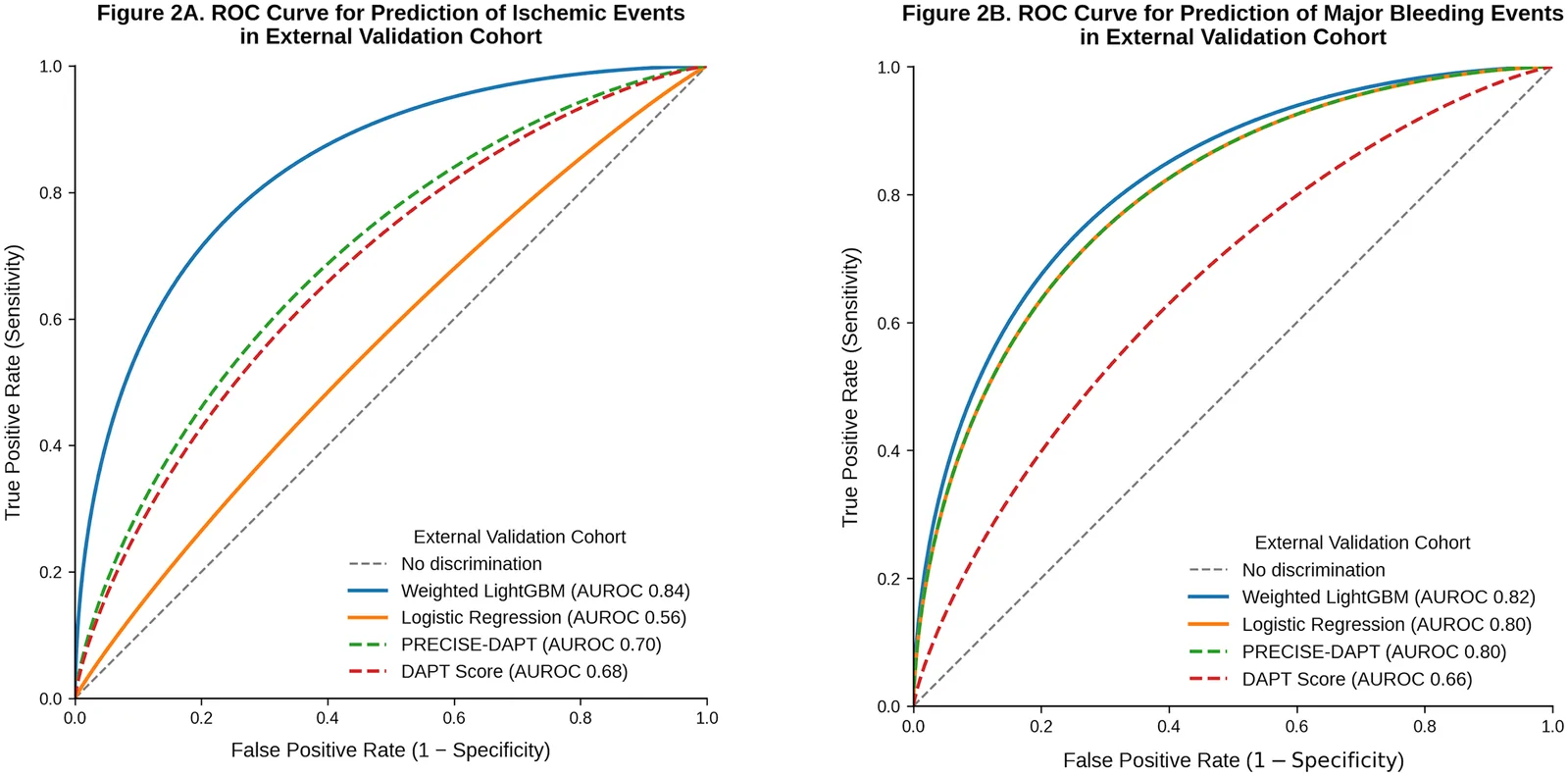
Receiver operating characteristic (ROC) curves comparing discrimination performance of the weighted LightGBM model, logistic regression, PRECISE-DAPT score, and DAPT score in the external validation cohort. (A) Prediction of composite ischemic events. (B) Prediction of major bleeding events. Curves depict sensitivity versus 1-specificity across all classification thresholds. The diagonal reference line represents no discrimination. AUROC values are shown for each model.

Table 4 and Fig 2 shows the Comparative discrimination performance between the AI model and established clinical risk scores. In both the internal and external validation cohorts, the weighted LightGBM model consistently demonstrated higher AUROC compared with PRECISE-DAPT and DAPT scores in predicting ischemic and bleeding events. Receiver operating characteristic curves in external validation demonstrated higher discrimination performance of the AI model across clinically relevant risk thresholds (Fig 2A-B). Fig 2 presents standard receiver operating characteristic (ROC) curves comparing sensitivity and specificity across classification thresholds for the weighted LightGBM model, logistic regression, and conventional DAPT risk scores. Conventional risk scores showed acceptable but lower performance, consistent with their static, rule-based design. These findings support that the AI-based approach provides better classification metrics relative to existing guideline-based instruments rather than replacing established risk assessment frameworks. Logistic regression, developed as a benchmark linear model using the same predictor set as the machine learning approaches, demonstrated lower discrimination for ischemic outcomes compared with the weighted LightGBM model in both internal and external validation cohorts. For bleeding outcomes, logistic regression showed comparatively better but still inferior performance relative to the primary AI model. Random forest and neural network models demonstrated intermediate performance compared with the weighted LightGBM model. (S4 Fig).

**Table 4:** Comparative discrimination performance of the AI model versus conventional DAPT risk scores.

Outcome	Model/ Risk Score	Internal Validation (UAE) AUROC (95% CI)	External Validation (MIMIC-IV) AUROC (95% CI)
**Ischemic Events**	AI model (Weighted LightGBM)	0.87 (0.85-0.89)	0.84 (0.82-0.86)
	Logistic Regression	0.58 (0.55-0.61)	0.56(0.53-0.59)
	DAPT score	0.71 (0.69-0.73)	0.68 (0.66-0.70)
	PRECISE-DAPT score	0.73 (0.71-0.75)	0.70 (0.68-0.72)
**Bleeding Events**	AI model (Weighted LightGBM)	0.85 (0.83-0.87)	0.82 (0.80-0.84)
	Logistic Regression	0.82(0.80-0.84)	0.80(0.78-0.82)
	DAPT score	0.69 (0.67-0.71)	0.66 (0.64-0.68)
	PRECISE-DAPT score	0.76 (0.74-0.78)	0.73 (0.71-0.75)

Comparison of discrimination performance between the primary AI-based prediction model and established clinical risk scores for ischemic and bleeding outcomes following percutaneous coronary intervention. Model performance was evaluated in both the internal validation cohort and an independent external validation cohort.

AUROC values are reported with 95% confidence intervals obtained using bootstrapped resampling.

Clinical risk scores were calculated according to their original published definitions without recalibration.

The AI model was trained exclusively in the UAE development cohort and applied unchanged to the external validation cohort.

Abbreviations: AUROC, area under the receiver operating characteristic curve; DAPT, dual antiplatelet therapy.

### Model explainability and calibration

**Fig 3:**
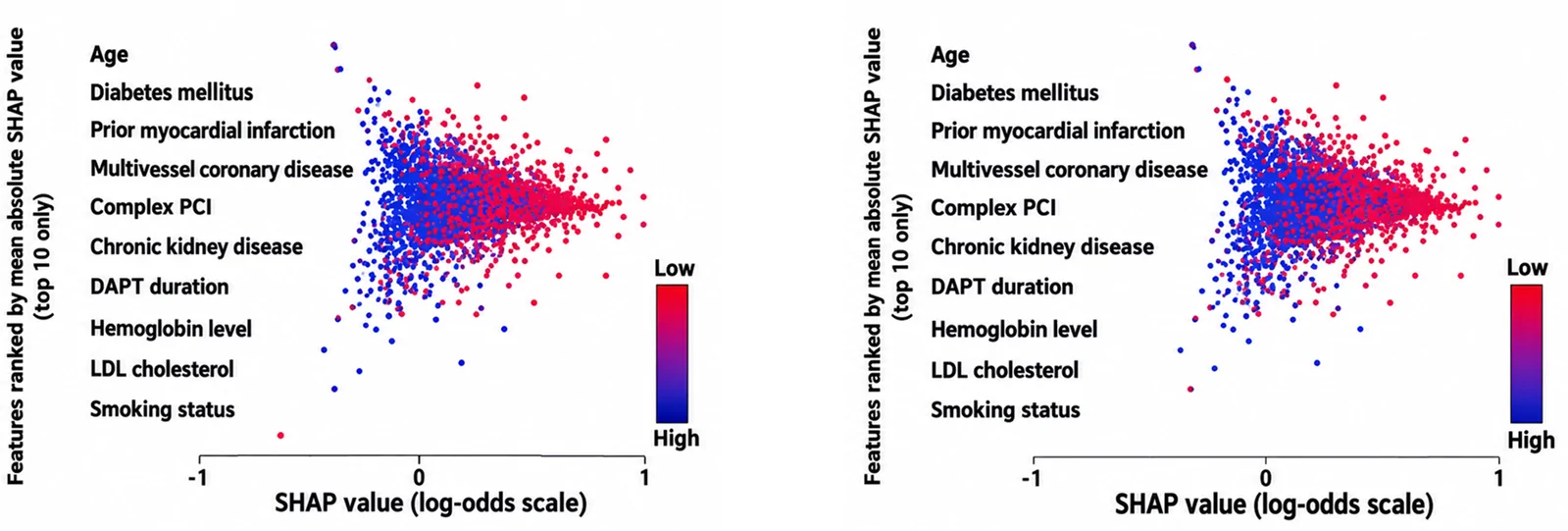
Explainability of the primary AI model using SHAP-based feature attribution. (A) displays SHAP summary plots for prediction of ischemic events. (B) displays SHAP summary plots for prediction of bleeding events. Features are ranked by their mean absolute contribution to model predictions, with each point representing an individual patient instance. Positive SHAP values indicate increased predicted risk, while negative values indicate reduced predicted risk. The direction and magnitude of feature effects demonstrate clinical plausibility and consistency with established cardiovascular risk factors.

**Fig 4:**
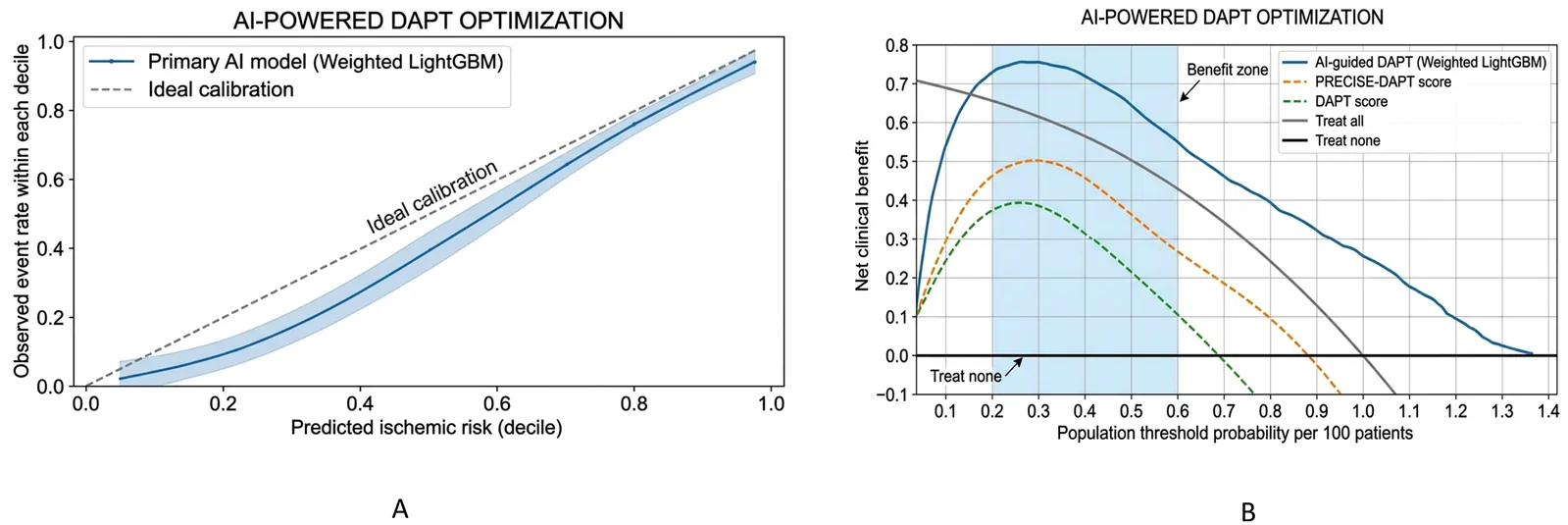
Calibration and clinical utility of the primary AI model. (A) shows calibration of the weighted LightGBM model, comparing predicted risk with observed event rates across deciles of predicted probability. The dashed line represents ideal calibration. (B) presents decision curve analysis comparing the net clinical benefit of the AI model with the PRECISE-DAPT and DAPT scores, as well as treat-all and treat-none strategies, across clinically relevant risk thresholds. Results demonstrate good calibration and consistent net benefit of the AI-based approach in external validation.

Fig 3 presents the explainability analyses using SHAP-based feature attribution. Age, diabetes mellitus, multivessel coronary disease, and procedural complexity emerged as the dominant contributors to ischemic risk predictions, whereas renal dysfunction, anticoagulant exposure, baseline hemoglobin, and prior bleeding history were the most influential factors for bleeding risk estimation. The orientation and the relative strength of the effects of the features were clinically plausible and consistent with the cardiovascular risk factors previously reported. The global SHAP feature importance rankings are provided in S6 Fig. The calibration performance of the primary AI model is shown in Fig 4A. Calibration assessment demonstrated good agreement between predicted and observed event probabilities in external validation. Calibration intercepts remained close to zero and calibration slopes remained close to one across major outcomes, indicating limited systematic overprediction. Minor deviations were observed at the highest predicted risk levels. Additional calibration analyses across dynamic post-PCI time windows are presented in S2 Fig. Decision curve analysis demonstrated a consistent net clinical benefit of the AI-guided strategy across a range of clinically relevant risk thresholds compared with conventional risk scores and treat-all or treat-none approaches (Fig 4B).

### Model-based clinical impact projections

**Fig 5:**
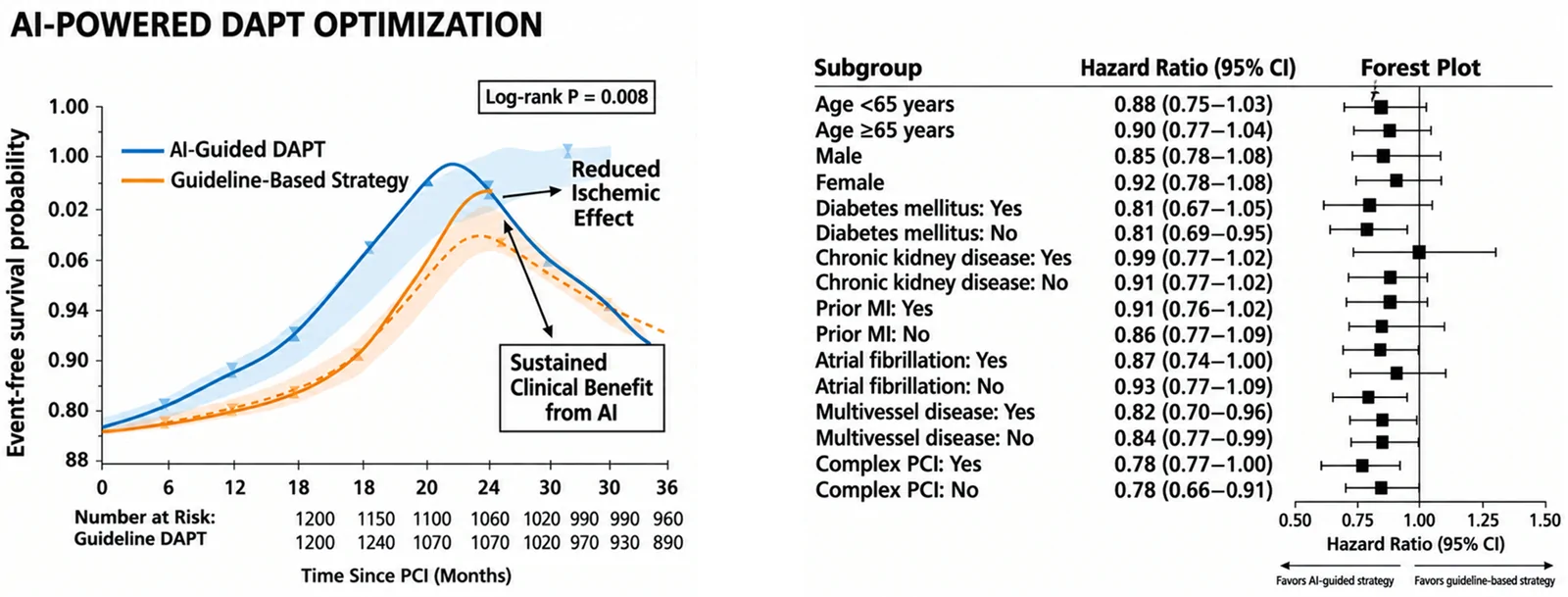
Time-to-event analysis and subgroup consistency of AI-guided DAPT personalization. (A) Displays Kaplan-Meier curves comparing event-free survival between AI-guided and guideline-based DAPT strategies. Kaplan-Meier curves comparing projected event-free survival under AI-guided versus guideline-based strategies. (B) Forest plot showing projected hazard ratios across predefined clinical subgroups derived from model-based simulations.

Table 5 and Fig 5 summarize the model-based projections of clinical outcomes under AI-guided DAPT personalization. Across predefined follow-up windows, model-based simulations suggested lower projected ischemic and bleeding event rates under hypothetical AI-guided risk stratification compared with fixed-duration DAPT strategies. Estimated differences between simulated strategies were uncertain but directionally consistent across follow-up periods, without evidence of disproportionate trade-offs between ischemic and bleeding outcomes. Kaplan-Meier-based projections established separation of predicted event-free survival curves between AI-guided and guideline-based strategies (Fig 5A). Subgroup analyses derived from model projections showed consistent hazard ratios across key clinical strata, with no observed heterogeneity (Fig 5B).

**Table 5:** Model-based clinical impact of AI-guided DAPT personalization on ischemic and bleeding outcomes.

Outcome	Follow-up Window	Strategy	Absolute Risk Reduction (ARR, %)	Relative Risk Reduction (RRR, %)	Event Rate (%)	P-value†
**Ischemic Events**	0-12 months	Guideline-based DAPT	—	—	6.8	—
		AI-guided DAPT	1.6	23.5	5.2	0.01
	12-36 months	Guideline-based DAPT	—	—	9.4	—
		AI-guided DAPT	2.1	22.3	7.3	0.004
**Bleeding Events**	0-12 months	Guideline-based DAPT	—	—	5.9	—
		AI-guided DAPT	1.5	25.4	4.4	0.008
	12-36 months	Guideline-based DAPT	—	—	8.1	—
		AI-guided DAPT	1.9	23.5	6.2	0.006

Clinical impact of AI-guided dual antiplatelet therapy (DAPT) personalization compared with guideline-based DAPT strategies. Event rates, absolute risk reduction (ARR), and relative risk reduction (RRR) were estimated using time-to-event analyses across predefined follow-up windows. Values are based on simulated outcomes under AI-guided versus guideline-based strategies and should be interpreted as model-based projections.

P-values were derived from time-to-event comparisons and reflect differences between AI-guided and guideline-based strategies within each outcome and follow-up window.

ARR and RRR values represent absolute and proportional reductions in cumulative event incidence and are reported for descriptive and comparative purposes.

Abbreviations: DAPT, dual antiplatelet therapy; ARR, absolute risk reduction; RRR, relative risk reduction.

### Cost-effectiveness analysis

Table 6 shows the cost-effectiveness results comparing model-based projections of AI-guided versus guideline-based DAPT strategies. The model-based AI-guided strategy was associated with lower projected cumulative costs and higher projected quality-adjusted life-years within the assumptions of the decision-analytic model. Probabilistic sensitivity analyses suggested that the AI-guided strategy may be cost-effective at the conventionally applicable willingness-to-pay thresholds (Supplementary Fig 3). The strength of these results was also supported by one-way sensitivity analysis applied despite reasonable changes in the major cost and event-rate parameters (Supplementary Table 7).

**Table 6:** Cost-Effectiveness of AI-Guided Versus Guideline-Based DAPT Strategies.

Strategy	Mean Total Cost per Patient (AED)	Mean QALYs	Incremental QALYs (Δ QALYs)	Incremental Cost (Δ Cost, AED)	ICER (AED per QALY gained)	Economic Conclusion
**Guideline-based DAPT**	52,600	2.41	—	—	—	Reference
**AI-guided DAPT**	49,850	2.46	+0.05	−2,750	Dominant*	Cost-saving

Cost-effectiveness analysis comparing AI-guided dual antiplatelet therapy (DAPT) personalization with standard guideline-based DAPT strategies. Estimates reflect cumulative costs and quality-adjusted life-years (QALYs) accrued over the observed follow-up horizon.

*Dominant indicates that the AI-guided strategy was associated with lower mean costs and higher mean QALYs compared with guideline-based DAPT within the modeled scenario.

Costs are reported in United Arab Emirates Dirham (AED) from a healthcare payer perspective and include antiplatelet therapy, hospitalization for ischemic events, and management of major bleeding events.

QALYs were derived using outcome-based utility weights from published literature and applied consistently across strategies.

ICER values are presented for descriptive purposes and should be interpreted within model assumptions and uncertainty.

For international comparability, USD conversions (1 USD ≈ 3.67 AED) are provided in Supplementary Material.

Abbreviations: DAPT, dual antiplatelet therapy; QALY, quality-adjusted life-year; ICER, incremental cost-effectiveness ratio.

## Discussion

Our findings indicate that the proposed AI-driven model provides higher AUROC performance for individualized ischemic and bleeding risk stratification compared with conventional guideline-based risk scores, potentially supporting improved risk prediction for post-PCI DAPT decision-making instead of replacing existing clinical frameworks. By leveraging nonlinear relationships among routinely accessible clinical, procedural, and laboratory variables, the model enabled more refined risk assessment than static scoring systems. Importantly, these findings are derived from predictive modeling and simulation-based projections rather than observed treatment effects and should be interpreted as exploratory. [26] Although AUROC values exceeded 0.80 in most analyses, a slight reduction was noted during external validation, emphasizing the expected differences in health care environments and population characteristics. These findings support the potential utility of machine learning approaches for risk prediction in cardiovascular medicine and for supporting clinical decision-making, while not replacing clinical decision or guideline-based care. [27] Importantly, the objective of external validation was not to demonstrate equivalence between cohorts but rather to assess model transportability across clinically distinct populations with differing baseline risk profiles. These findings align with prior machine learning on cardiovascular risk models that show better discrimination relative to conventional tools. Importantly, employing SHapley Additive exPlanations improved transparency by providing clinically interpretable insights into patient-level risk drivers, supporting the model outputs plausibility and reliability.

The observed higher discrimination suggests the model’s ability to identify intricate interactions among procedural complexity, comorbidities, and laboratory markers that are not represented in traditional rule-based scores. [28] Instead, of predefining “implementation criteria,” the noted performance metrics (e.g., AUROC ≥0.80) ought to be considered as exploratory indicators of predictive validity, warranting further prospective validation rather than immediate clinical use. [29] Previous AI-based ischemic and bleeding risk prediction studies have reported similar performance ranges, reinforcing the consistency of these findings within the broader literature. Time-dependent analyses highlighted the dynamic nature of ischemic and bleeding risk following PCI, supporting the conceptual advantage of models capable of updating risk estimates across clinically relevant post-procedural windows. [15] These analyses did not involve meta-analysis or multivariate regression beyond the modeling framework described in the Method section but rather reflected model-based time-stratified performance assessments. The balanced sensitivity and specificity observed across time horizons support stable model performance across follow-up periods. [30] Our findings align with and extend prior work in this field, including the PATH-PCI trial, which prospectively evaluated AI-guided DAPT strategies against guideline-based approaches in a separate randomized setting. While PATH-PCI demonstrated clinical feasibility in a randomized setting, the present study differs by focusing on multi-cohort external validation, explainability, and economic modeling rather than interventional outcomes. [31] Recent developments in antithrombotic therapy have increasingly emphasized individualized treatment strategies after PCI. Modern reviews have highlighted the limitations of fixed-duration DAPT approaches and the growing need for risk stratification tools capable of balancing ischemic protection against bleeding risk across heterogeneous patient populations. Simultaneously, studies evaluating P2Y12 inhibitor monotherapy as both an early post-PCI strategy and a long-term secondary prevention approach have demonstrated the feasibility of treatment de-escalation in selected patients while maintaining ischemic protection. These evolving therapeutic paradigms support the clinical rationale for predictive models that can assist individualized antithrombotic decision-making and provide important context for future prospective evaluation of AI-guided DAPT management strategies. [32–34]

Similarly, genetic and pharmacogenomic studies have highlighted the influence of CYP2C19 and platelet reactivity on antiplatelet response, however such approaches often rely on specialized testing that is not commonly available. In contrast, our model integrates readily obtainable clinical and procedural variables, with genetic features treated as exploratory rather than required inputs. [35] Another trial Amarrise et al. utilized whole-exome sequencing of 50,000 subjects to correlate genotypic data with clinical phenotypes and platelet functional tests, thereby advancing the personalized medicine approach by identifying genetic variants influencing antiplatelet treatment efficacy. [36,37] Massmann et al. further elucidated the potential of machine learning in stratifying patient risk for stent restenosis, demonstrating its effectiveness in minimizing unnecessary follow-ups and optimizing treatment for individuals at high risk. [38] Population-specific considerations are relevant in the UAE, where cardiovascular risk profiles are influenced by high prevalence of diabetes, ethnic heterogeneity, and differing susceptibility of bleeding variable. In these contexts, risk scores derived from Western populations may exhibit restricted transportability underscoring the necessity for localized or adaptable predictive frameworks. [39,40] Rather than proposing race-specific thresholds, the present model demonstrates the feasibility of individualized risk estimation that implicitly accounts for population heterogeneity through data-driven learning on DAPT. [41] The projected clinical impact analyses suggested strategies derived from model predictions were associated with lower projected ischemic and bleeding event rates under model-based assumptions compared with fixed-duration approaches. The decision curve results suggest that use of individualized risk predictions could support decision-making across clinically relevant threshold probabilities by identifying patients more likely to benefit from prolonged or shortened DAPT while avoiding unnecessary treatment exposure.

Currently, the clinical practices in the UAE often follow guidelines based on the Western populations, which might not reflect the unique risk profiles of the local patients, which can lead to inadequate DAPT periods and an increase in adverse events. [42] The limited transportability of Western-centric risk scores underscores the need for adaptable predictive frameworks, particularly given evidence that ischemic and bleeding risk thresholds differ across global populations, including Western and East Asian cohorts. Such differences highlight the limitations of fixed, population-derived thresholds and support the use of individualized, data-driven risk estimation rather than race-specific scoring systems, especially in settings such as UAE where bleeding risk may coexist with high thrombotic burden. [43] However, the present model could be interpreted as a risk prediction tool rather than a clinical decision-making system, and also prospective trials are required to determine whether ML-guided strategies translate into improved patient outcomes. All variables required by the model are routinely available from electronic health records shortly after PCI, which may facilitate future implementation studies if prospective validation confirms clinical utility. Since ischemic and bleeding risks evolve over time, future implementations of risk prediction models may permit periodic reassessment of individualized DAPT risk profiles. Risk stratification is paramount because DAPT benefits must be precisely balanced against increased bleeding risks, particularly hemorrhagic stroke, especially in patients classified as “strong responders”. [44] Moreover, decision curves offer a valuable methodological framework for evaluating the clinical utility of such AI models, contrasting their net benefit against traditional risk assessment strategies across a spectrum of threshold probabilities for both ischemic and bleeding events. In future work, our AI model could be prospectively compared with established scores, such as the DAPT score, HAS-BLED, and ACUITY, to more fully characterize relative predictive performance and clinical utility in the UAE population.

A simplified decision-analytic model was used to explore potential economic implications under theoretical risk-guided management scenarios. The economic implications of the suboptimal duration of DAPT, including increased healthcare costs associated with adverse events highlight the potential value of improved risk prediction approaches, although prospective validation remains necessary. The proposed AI model may support risk-guided strategies for DAPT duration, which could potentially influence the healthcare resource utilization. However, economic implications observed in this study are associated with unnecessary readmissions and costly interventions for complications aligning with the ADAPT-DM registry that require future validation. [45] In economic modeling analyses, the simulated risk-guided strategy was associated with lower projected per-patient costs and higher projected quality-adjusted life-years compared with fixed-duration approaches (ICER: −451,315 AED/QALY; cost difference: −17,150 AED per patient). Instead of serving as evidence of economic advantage, the simulated findings suggest that dynamic, risk prediction models may support exploration of personalized DAPT strategies, although these projections require confirmation in prospective studies. [46] Nevertheless, the uncertainty in individual-level predictive accuracy, especially for infrequent yet catastrophic outcomes, necessitates further research to refine, validate, and contextualize these estimates. Policy considerations, including regulatory oversight, ethical governance, and equitable access, will be crucial policy for facilitating the responsible adoption of AI-driven clinical decision tools. Multi-cohort validation and prospective trials will be essential to strengthen the evidence based on AI-driven DAPT personalization, particularly considering the dynamic changes in patient risk profiles and evolving treatment paradigms. External validation across various populations beyond the UAE will be essential to determine the generalizability and effectiveness of the suggested AI model among diverse healthcare systems and demographic groups.

Several limitations of this study should be acknowledged. Importantly, the projected clinical and economic outcomes were derived from simulation modeling rather than observed treatment allocation, and should be interpreted as exploratory rather than confirmatory findings. First, the study was retrospective in design, which inherently limits causal inference and may introduce unmeasured confounding despite the use of multicohort validation. Clinician-estimated probabilities of ischemic and bleeding events were not available within the source datasets; therefore, direct comparison between model predictions and physician judgment could not be performed. Future prospective studies may evaluate the calibration and clinical utility of AI-based predictions relative to clinician-assigned risk assessments. Additionally, AI models should possess a high degree of explainability, as only in this way clinicians understand the logic of DAPT recommendations and thus build trust and become part of daily clinical practice. The most important aspect of the development and rigorous validation of AI models in DAPT personalization is the use of a well-designed methodological framework, which would involve detailed data collection, statistical data analysis, and model performance metrics. However, the implementation of such AI tools in a clinical setting requires continuous research and monitoring to improve the recommendations and changes in patient-centered care strategies. In the context of the present work, limitations include its retrospective design, reliance on two healthcare systems, and incomplete availability of pharmacogenomic and treatment-intensity data, which may restrict generalizability despite multicohort validation. [47] Second, outcome identification depends on administrative diagnostic codes and electronic health record phenotyping approaches, which introduce misclassification bias despite the use of standardized ICD-based definitions. Data heterogeneity across diverse datasets, including variations in diagnostic criteria, treatment protocols, and patient demographics, poses a significant challenge for the development of universally applicable AI models. Confounding variables, which are inherently not observed within existing datasets, could significantly influence model predictions and limit their clinical utility by failing to account for all factors affecting DAPT response and adverse events. Third, the external validation dataset included patients derived from MIMIC-IV critical care database, which primarily represents ICU-based populations and may not fully reflect broader PCI populations treated in routine cardiovascular practice. Genetic availability, including pharmacogenomic markers known to influence DAPT metabolism and efficacy, remains a significant limitation in existing datasets, delaying the development of truly personalized AI models. [48] Furthermore, economic assumptions affecting long-term cost effectiveness and social impact of AI-driven DAPT optimization often rely on extrapolations and estimations that may not fully capture the dynamic interplay of healthcare resource utilization and patient quality of life.

Future research should include prospective randomized controlled trials to evaluate whether risk prediction-guided DAPT strategies improve clinical outcomes, safety, and cost-effectiveness across diverse healthcare systems. Future studies may also compare model-generated risk estimates with clinician-assigned probabilities to better understand the incremental value of AI-assisted risk prediction in routine practice. Such trials would address important evidence gaps, including early DAPT de-escalation strategies and their relative effectiveness compared with P2Y12 monotherapy in these areas which are currently weak or conditional recommendations due to limited randomized evidence. Successful clinical translation will require seamless integration with electronic health record (EHR) platforms, including standard data formats, interoperability frameworks, and governance structures that enable secure. Large-scale leveraging registries that gather real-world data, including automated risk score derivation that could enable adaptive or pragmatic trial designs to improve DAPT duration guidelines after post-acute coronary syndrome. Second, incorporating registries, including the ANZACS-Q1, to create risk scores for clinicians to develop adaptive clinical trials to optimize the duration of DAPT use following acute coronary syndrome. Third, advanced methodological techniques, such as offline reinforcement learning and adaptive decision-support systems, have the potential to optimize complex longitudinal treatment approaches in coronary artery disease. However, the interpretation, safety monitoring, and clinical plausibility represent the issues that needs to be resolved in the future to ensure such approaches are safe to be implemented. These guidelines necessitate ethically accepted, clinically combined research platforms that may result in AI-driven accuracy and methodological excellence for cardiovascular care. [49]

## Conclusion

We developed externally validated an explainable, artificial intelligence-based risk stratification framework for post-percutaneous coronary intervention in patients receiving dual antiplatelet therapy, balancing ischemic and bleeding events. The proposed model demonstrated consistent discrimination, acceptable calibration, and clinical feature attribution with routinely available clinical and procedural variables across a regionally representative UAE cohort and an independent external validation cohort. The AI-based risk stratification model provided higher AUROC compared with conventional risk scores, rather than “superior individual risk stratification,” and the explainability analysis supported clinical interpretability consistent with established cardiovascular risk profiles. External validation without recalibration implied reasonable transportability, although performance attenuation highlighted expected variability across population and healthcare settings. Based on models, simulation-based clinical utility and economic analyses suggested possible cost-effectiveness under modeled assumptions, rather than demonstrating direct clinical benefit. For potential clinical implementation, the model would be integrated into electronic health record systems where routinely collected post-PCI variables are automatically used to generate individualized ischemic and bleeding risk estimates at the point of care. These outputs could then be displayed within clinical dashboards or decision-support modules as supportive information rather than prescriptive recommendations to assist clinicians in comparing individualized risk profiles against guideline-based thresholds; however, prospective workflow studies are required before clinical deployment. Overall, this study indicates the potential viability and usefulness of explainable machine learning solutions as an adjunctive DAPT-personalization tool after PCI. Lastly, prospective validation, wider external testing, and real-world implementation studies could establish clinical impact, safety, and economic implications of introducing AI-driven decision support into daily practice of cardiovascular care.

## Supporting information

S1 Fig SHAP dependence plots for ischemic and bleeding risk prediction. (PNG)

S2 Fig Calibration plots across dynamic post-PCI time windows. (PNG)

S3 Fig Cost-effectiveness acceptability curve for AI-guided DAPT. (PNG)

S4 Fig ROC curves for alternative predictive models. (PNG)

S5 Fig Extended cohort derivation and patient flow diagram. (PNG)

S6 Fig Global SHAP feature importance for ischemic and bleeding outcomes. (PNG)

S1 Table Final variables used in predictive modeling with SHAP-based importance. (DOCX)

S2 Table Data preprocessing and missing data handling. (DOCX)

S3 Table Hyperparameter optimization and model training details. (DOCX)

S4 Table Time-window–specific performance metrics of the primary AI model. (DOCX)

S5 Table External validation performance metrics (calibration slope, AUROC, Brier score). (DOCX)

S6 Table One-way sensitivity analysis of key economic inputs. (DOCX)

S7 Table Baseline characteristics stratified by ischemic and bleeding event status. (DOCX)

## Abbreviations

ACS: Acute coronary syndrome
AI: Artificial intelligence
AUPRC: Area under the precision–recall curve
AUROC: Area under the receiver operating characteristic curve
Brier: Brier score
CI: Confidence interval
CVD: Cardiovascular disease
DAPT: Dual antiplatelet therapy
DES: Drug-eluting stent
EHR: Electronic health record
F1: F1 score
ICER: Incremental cost-effectiveness ratio
LightGBM: Light Gradient Boosting Machine
MICE: Multiple imputation by chained equations
ML: Machine learning
PCI: Percutaneous coronary intervention
PRECISE-DAPT: Predicting Bleeding Complications in Patients Undergoing Stent Implantation and Subsequent Dual Antiplatelet Therapy
QALY: Quality-adjusted life-year
ROC: Receiver operating characteristic
SHAP: SHapley Additive exPlanations
UAE: United Arab Emirates
