# Supplementary material for: A machine learning model for predicting ischemic and bleeding risk after percutaneous coronary intervention: Development and external validation
Source: PLoS One. 2026 Sep 15;21(9):e0353066. doi: 10.1371/journal.pone.0353066 (PMC13577505; doi:10.1371/journal.pone.0353066)
Supplement: Supplementary file 1 [file pone.0353066.s007.docx]

**Supplementary Table S1-**Clinical Outcome Definitions and ICD Code Mapping

| **Outcome** | **Definition** | **ICD-9 Codes** | **ICD-10 Codes** |
| --- | --- | --- | --- |
| Ischemic stroke | Acute ischemic cerebrovascular event | 433.x, 434.x | I63.x |
| Stent thrombosis | PCI-related stent thrombosis | 996.72 | T82.8 |
| Myocardial infarction | Acute MI during follow-up | 410.x | I21.x |
| Major bleeding (BARC 3/5 proxy) | Hospitalized bleeding event requiring intervention or transfusion | 578.x, 459.0 | K92.2, I85.0, I86.4 |
| In-hospital bleeding | Bleeding event during index hospitalization | same codes | same codes |
