# Supplementary material for: A machine learning model for predicting ischemic and bleeding risk after percutaneous coronary intervention: Development and external validation
Source: PLoS One. 2026 Sep 15;21(9):e0353066. doi: 10.1371/journal.pone.0353066 (PMC13577505; doi:10.1371/journal.pone.0353066)
Supplement: Supplementary file 2 [file pone.0353066.s008.docx]

**Supplementary Table S2-** Final Variables Used in Predictive Modeling (Ischemia and Bleeding Risk after PCI).

**Legend**

Both: Feature included in both ischemic and bleeding prediction models

Derived: Calculated from primary clinical variables

| **Variable Category** | **Variable Name** | **Used for Task** | **Type** | **SHAP Score / Rank** | **Notes** |
| --- | --- | --- | --- | --- | --- |
| **Demographics** | Age | Both | Continuous | 0.71 (Rank #5 Bleeding) | Age at time of PCI |
|  | Sex | Both | Binary | — | Male/Female |
|  | BMI | Both | Continuous | 0.63 (Rank #6 Ischemia) | Body mass index (kg/m²) |
| **Comorbidities** | Diabetes Mellitus | Both | Binary | 0.72 (Rank #4 Ischemia) | Based on ICD-10 codes or EHR diagnosis |
|  | Hypertension | Both | Binary | 0.65 (Rank #7 Bleeding) | Confirmed diagnosis or antihypertensive use |
|  | Dyslipidemia | Ischemia | Binary | 0.71 (Rank #5 Bleeding) | On lipid-lowering therapy or elevated LDL |
|  | Prior MI | Ischemia | Binary | 0.79 (Rank #2 Ischemia) | Documented history |
|  | Prior Stroke | Bleeding | Binary | 0.60 (Rank #8 Bleeding) | Non-hemorrhagic or hemorrhagic stroke |
|  | Anemia | Bleeding | Binary | 0.67 (Rank #3) | Hemoglobin <13g/dL (men), <12g/dL (women) |
|  | Chronic Kidney Disease | Bleeding | Binary | 0.60 (Rank #8 Bleeding) | eGFR < 60 ml/min/1.73 m² |
| **PCI Characteristics** | Multivessel Disease | Ischemia | Binary |  | ≥2 vessels treated |
|  | Stent Type (DES vs. BMS) | Both | Categorical | — | Includes limitation: some BMS data |
|  | Overlapping Stents | Ischemia | Binary | — | Yes/No |
| **Medications at Discharge** | Beta Blockers | Both | Binary | 0.50 (Rank #12) | Guideline-recommended for post-ACS |
|  | Statins | Ischemia | Binary | 0.57 (Rank #9) | High/moderate intensity statin use |
|  | PPIs | Bleeding | Binary | 0.51 (Rank #11) | Proton pump inhibitors use |
|  | Oral Anticoagulants | Bleeding | Binary | 0.76 (Rank #2 Bleeding) | Warfarin, DOACs |
|  | Triple Therapy (OAC + DAPT) | Bleeding | Binary | 0.85 (Rank #1 Bleeding) | High-risk antithrombotic combination |
| **Laboratory Values** | Platelet Count | Bleeding | Continuous | 0.59 (Rank #9 Bleeding) | From index hospitalization |
|  | Hemoglobin | Bleeding | Continuous | 0.55 (Rank #10 Bleeding) | From index hospitalization |
|  | Creatinine | Both | Continuous | 0.73 (Rank #3 Ischemia) | Used to calculate eGFR |
| **Genetic Data** | CYP2C19 LOF Allele | Ischemia | Binary | 0.83 (Rank #1 Bleeding) | LOF allele presence |
| **Procedure Context** | ACS vs. CCS | Both | Categorical | 0.62 (Rank #5 Ischemia) | Stratified analysis in model |
|  | In-Hospital Bleed | Bleeding | Binary | 0.59 (Rank #9 Bleeding) | Before discharge |
| **DAPT Characteristics** | Initial DAPT Regimen | Both | Categorical | 0.55 (Rank #10 Bleeding) | Clopidogrel vs. Prasugrel/Ticagrelor |
|  | Planned DAPT Duration | Both | Categorical | 0.73 (Rank #3 Ischemia) | <6, 6–12, >12 months |

**Footnotes*** SHAP (Shapley Additive Explanations) values represent each variable’s relative contribution to AI model predictions for ischemic and bleeding risks. Variables are ranked independently for both outcomes. Variables such as anemia, CKD, beta blocker use, and proton-pump inhibitors (PPIs) were retained based on consistent influence across subgroups. Feature importance is derived from the optimized LightGBM model validated on cross-population datasets.
