# Supplementary material for: A machine learning model for predicting ischemic and bleeding risk after percutaneous coronary intervention: Development and external validation
Source: PLoS One. 2026 Sep 15;21(9):e0353066. doi: 10.1371/journal.pone.0353066 (PMC13577505; doi:10.1371/journal.pone.0353066)
Supplement: Supplementary file 3 [file pone.0353066.s009.docx]

**Supplementary Table S3-**Data Preprocessing and Missing Data Handling

**Legend**

- **MICE**: Multivariate Imputation by Chained Equations
- **Complete-case**: Excluded only if key procedural variables missing
- **Missing indicator**: Binary indicator added to model to preserve sample size

| **Variable Group** | **Representative Variables** | **Imputation Method** | **Missing (%) UAE Development** | **Missing (%) External Validation** | **Sensitivity Analysis Performed** |
| --- | --- | --- | --- | --- | --- |
| **Demographics** | Age, Sex | None (complete-case) | <1% | <1% | No |
| **Anthropometrics** | Body mass index | Median imputation | 6–8% | 10–12% | Yes |
| **Comorbidities** | Diabetes, Hypertension, CKD, AF | Assumed absent if missing | 3–5% | 5–7% | Yes |
| **Prior Clinical History** | Prior MI, Prior bleeding | Assumed absent if missing | 4–6% | 6–8% | Yes |
| **Laboratory Parameters** | Hemoglobin, Platelets, Creatinine | MICE (multivariable) | 8–12% | 12–18% | Yes |
| **Procedural Characteristics** | Multivessel disease, Complex PCI | Complete-case | <3% | 4–6% | Yes |
| **Medication Exposure** | Anticoagulant use, P2Y12 type | Categorical mode | 2–4% | 5–7% | Yes |
| **Renal Function (Derived)** | eGFR | Derived from creatinine | 0%* | 0%* | No |
| **DAPT Strategy Variables** | Baseline DAPT duration | Complete-case | <2% | NA† | No |
| **Genetic Variables** | CYP2C19 variants | Missing indicator | 15–20% | NA† | Yes |
| **Outcome Variables** | Ischemic, Bleeding events | Adjudicated outcomes | 0% | 0% | No |

**Footnotes**

* Estimated glomerular filtration rate (eGFR) was derived from serum creatinine using standard equations and therefore did not require separate imputation.

† Genetic variables and baseline DAPT duration were not available in the external validation cohort and were excluded from external model evaluation, consistent with the Methods.

Sensitivity analyses included:

- Complete-case reanalysis
- Alternative imputation strategies (median vs MICE)
- Exclusion of genetic variables
  Results were directionally consistent with the primary analysis.
