# Supplementary material for: A machine learning model for predicting ischemic and bleeding risk after percutaneous coronary intervention: Development and external validation
Source: PLoS One. 2026 Sep 15;21(9):e0353066. doi: 10.1371/journal.pone.0353066 (PMC13577505; doi:10.1371/journal.pone.0353066)
Supplement: Supplementary file 4 [file pone.0353066.s010.docx]

**Supplementary Table S4-Hyperparameter Optimization and Model Training Details**

**Legend**

- **CV**: Cross-validation
- **p**: Number of input features
- **Class weighting** used to address outcome imbalance without synthetic oversampling
- **Temporal preservation** ensured no future information leaked into training folds.

| **Model** | **Optimization Method** | **Key Hyperparameters (Final Values / Search Ranges)** |
| --- | --- | --- |
| **Weighted LightGBM (Primary Model)** |  | • Number of estimators: **400** (search: 300–600) |
| • Learning rate: **0.05** (search: 0.01-0.10) |  |  |
| • Max depth: **8** (search: 6–10) |  |  |
| • Num leaves: **48** (search: 31-64) |  |  |
| • Feature fraction: **0.80** (search: 0.70-0.90) |  |  |
| • Bagging fraction: **0.80** (search: 0.70-0.90) |  |  |
| • Min data in leaf: **30** (search: 20-50) |  |  |
| • Class weight: **Inverse outcome frequency** | 5-fold stratified CV on UAE development cohort; folds preserved temporal order | Bayesian optimization (Tree-structured Parzen Estimator, 50 iterations) |
| **Random Forest** |  | • Number of trees: **800** (search: 500-1,000) |
| • Max depth: **15** (search: 10-20) |  |  |
| • Min samples per leaf: **10** (search: 5-20) |  |  |
| • Max features: **√p** |  |  |
| • Bootstrap sampling: **Enabled** | 5-fold stratified CV on UAE development cohort | Grid search |
| **Neural Network (Feed-Forward DNN)** |  | • Hidden layers: **2** |
| • Neurons per layer: **128 → 64** |  |  |
| • Activation function: **ReLU** |  |  |
| • Dropout rate: **0.30** |  |  |
| • Optimizer: **Adam** |  |  |
| • Learning rate: **0.001** |  |  |
| • Batch size: **64** |  |  |
| • Epochs: **50 (early stopping at patience = 5)** | 5-fold stratified CV with early stopping on validation folds | Random search (initial) + grid refinement |
| **Logistic Regression (Baseline)** |  | • Penalty: **L2** |
| • Regularization strength (C): **0.10** (search: 0.01–1.0) |  |  |
| • Solver: **liblinear** |  |  |
| • Class weight: **balanced** | 5-fold stratified CV | Grid search |

**Footnotes**

1. All hyperparameter optimization was conducted exclusively within the development cohort, with tuning nested inside cross-validation to prevent information leakage.
2. Final model selection prioritized calibration stability, external validation performance, and clinical plausibility, not AUROC alone.
3. External validation on the MIMIC-IV cohort used the finalized models without further tuning.
