# Supplementary material for: A machine learning model for predicting ischemic and bleeding risk after percutaneous coronary intervention: Development and external validation
Source: PLoS One. 2026 Sep 15;21(9):e0353066. doi: 10.1371/journal.pone.0353066 (PMC13577505; doi:10.1371/journal.pone.0353066)
Supplement: Supplementary file 5 [file pone.0353066.s011.docx]

**Supplementary Table S5-Time-Window–Specific Performance Metrics of the Primary AI Model**

**Legend**

- **AUROC**: Area under the receiver operating characteristic curve
- **AUPRC**: Area under the precision–recall curve
- Metrics derived from out-of-sample predictions only

| **Time Window (Months)** | **Outcome** | **AUPRC** | **AUROC** | **Specificity** | **Sensitivity** |
| --- | --- | --- | --- | --- | --- |
| **0–3** | Ischemia | 0.41 | 0.83 | 0.78 | 0.76 |
|  | Bleeding | 0.38 | 0.81 | 0.77 | 0.74 |
| **3–6** | Ischemia | 0.45 | 0.85 | 0.80 | 0.78 |
|  | Bleeding | 0.42 | 0.83 | 0.79 | 0.76 |
| **6–12** | Ischemia | 0.49 | 0.87 | 0.82 | 0.80 |
|  | Bleeding | 0.46 | 0.85 | 0.81 | 0.78 |
| **12–36** | Ischemia | 0.47 | 0.86 | 0.81 | 0.79 |
|  | Bleeding | 0.44 | 0.84 | 0.80 | 0.77 |

**Footnote**

1. Time windows were prespecified in the Methods and correspond to clinically meaningful post-PCI phases.
2. Performance metrics reflect evaluation of the primary weighted LightGBM model trained on the UAE development cohort.
3. External validation performance followed similar temporal trends and is summarized separately (Supplementary Table S5).
4. Slightly lower AUPRC values in early windows reflect lower event prevalence, not reduced model discrimination.
