# Supplementary material for: A machine learning model for predicting ischemic and bleeding risk after percutaneous coronary intervention: Development and external validation
Source: PLoS One. 2026 Sep 15;21(9):e0353066. doi: 10.1371/journal.pone.0353066 (PMC13577505; doi:10.1371/journal.pone.0353066)
Supplement: Supplementary file 6 [file pone.0353066.s012.docx]

**Supplementary Table S6-External Validation Performance Stratified by Outcome**

**Legend**

- **AUROC**: Area under the receiver operating characteristic curve
- **Calibration slope**: Agreement between predicted and observed risk (ideal = 1.0)
- **Brier score**: Mean squared error of probabilistic predictions (lower values indicate better calibration).

| **Outcome** | **Calibration Slope** | **AUROC** | **Brier Score** |
| --- | --- | --- | --- |
| **Ischemic events** | 0.94 | 0.82 | 0.108 |
| **Bleeding events** | 0.91 | 0.80 | 0.114 |

**Footnote**

1. External validation was performed using the MIMIC-IV cohort, which was not used in any phase of model training, hyperparameter tuning, or feature selection.
2. Performance metrics were calculated using out-of-sample predictions only, applying the finalized model trained on the UAE development cohort.
3. Calibration slopes close to unity indicate preserved risk ranking and minimal overfitting despite population and healthcare system differences.
4. Slightly lower AUROC and higher Brier scores compared with internal validation are expected and reflect the heterogeneity of the external cohort.
