# Supplementary material for: A machine learning model for predicting ischemic and bleeding risk after percutaneous coronary intervention: Development and external validation
Source: PLoS One. 2026 Sep 15;21(9):e0353066. doi: 10.1371/journal.pone.0353066 (PMC13577505; doi:10.1371/journal.pone.0353066)
Supplement: Supplementary file 7 [file pone.0353066.s013.docx]

**Supplementary Table S7-One-Way Sensitivity Analysis of Key Economic Inputs**

**Legend**

- **ICER**: Incremental cost-effectiveness ratio
- **Dominant**: Lower cost and higher effectiveness compared with guideline-based DAPT

| **Parameter Varied** | **Base-Case Assumption** | **Range Tested (±20%)** | **Dominance Retained*** | **Resulting ICER (USD/QALY)** |
| --- | --- | --- | --- | --- |
| **Cost of DAPT medications** | 1,200 USD | 960 – 1,440 | Yes | Dominant to 4,800 |
| **Cost of ischemic event hospitalization** | 9,500 USD | 7,600 – 11,400 | Yes | Dominant |
| **Cost of bleeding event management** | 4,200 USD | 3,360 – 5,040 | Yes | Dominant |
| **Ischemic event rate reduction (AI strategy)** | −2.1% | −1.7% to −2.5% | Yes | Dominant to 6,200 |
| **Bleeding event rate reduction (AI strategy)** | −1.6% | −1.3% to −1.9% | Yes | Dominant |
| **Utility decrement per ischemic event** | −0.08 QALY | −0.06 to −0.10 | Yes | Dominant to 5,400 |
| **Utility decrement per bleeding event** | −0.04 QALY | −0.03 to −0.05 | Yes | Dominant |
| **Time horizon** | 37 months | 24 – 48 months | Yes | Dominant to 7,100 |

**Footnote**

* Dominance retained indicates that the AI-guided DAPT strategy remained either cost-saving or cost-effective below commonly accepted willingness-to-pay thresholds (≤50,000 USD/QALY) across all tested scenarios.

Sensitivity analyses were conducted as one-way deterministic analyses, varying one parameter at a time while holding others constant. These results complement the probabilistic sensitivity analysis presented in Supplementary Figure S3.
