# Supplementary material for: A machine learning model for predicting ischemic and bleeding risk after percutaneous coronary intervention: Development and external validation
Source: PLoS One. 2026 Sep 15;21(9):e0353066. doi: 10.1371/journal.pone.0353066 (PMC13577505; doi:10.1371/journal.pone.0353066)
Supplement: Supplementary file 9 [file pone.0353066.s015.docx]

# **RESPONSE TO EDITOR AND REVIEWERS**

## Manuscript Title: **AI-driven personalization of dual antiplatelet therapy duration after PCI: Development and external validation of a machine learning model with economic evaluation**

# RESPONSE TO EDITOR

## Editorial Comment 1: Code Sharing Compliance

**Comment:**
PLOS ONE requires all author-generated code underpinning the findings to be made publicly available without restrictions upon publication.

**Response:**
We thank the Editor for highlighting the code sharing requirements. In compliance with PLOS ONE data and software availability policies, we have made all author-generated code publicly available in a dedicated GitHub repository:

**GitHub Repository (Public):**
<https://github.com/H123-lab/AI_Driven-DAPT-Personlization-Prediction>

The repository includes:

- Data preprocessing scripts
- Model development code (LightGBM, logistic regression, random forest, neural network)
- Feature engineering pipeline
- Risk stratification algorithms
- Visualization scripts
- Output generation workflows

All code is freely accessible and released without restrictions to ensure full reproducibility and reuse.

## Editorial Comment 2-5: Data Availability and Ethical Restrictions

**Comment:**
All data underlying the findings must be freely available or properly justified if restricted.

**Response:**
We have revised the Data Availability Statement to fully comply with journal policy while respecting ethical and institutional constraints.

**Updated Data Availability Statement (Manuscript):**

The data supporting the findings of this study are available within the article and its Supplementary Materials. The analytical code, model development workflow, and documentation are publicly available in the GitHub repository (<https://github.com/H123-lab/AI_Driven-DAPT-Personlization-Prediction>) to ensure transparency and reproducibility.

The MIMIC-IV database is publicly accessible to qualified researchers through the PhysioNet Credentialed Health Data Access framework (<https://physionet.org/>) following completion of mandatory training and approval.

The UAE clinical dataset used for external validation contains de-identified patient-level health records obtained under institutional data governance policies and ethical restrictions. Due to these regulatory and privacy constraints, the dataset cannot be publicly shared.

Requests for access to the UAE dataset may be directed to the corresponding author, who will liaise with the relevant institutional data custodians. Access is subject to institutional review board approval and data-sharing policies.

## Editorial Comment 5: Data Access Contact Information Requirement

**Response:**

We confirm that appropriate data access governance has been clarified as follows:

The UAE clinical dataset is governed by institutional data governance and ethical oversight frameworks. Requests for access should be directed to the corresponding author, who will coordinate with institutional data custodians. Access is subject to institutional approval and privacy regulations. The MIMIC-IV dataset is available via PhysioNet credentialed access, and qualified researchers may directly obtain access through standard certification procedures.

This information has been included in both the manuscript Method section in ethics statement and the submission response form.

**RESPONSE TO REVIEWER #1 Highlight in 🟢**

**Comment 1: Insufficient model development detail (variables, missing data, hyperparameter tuning, explainability)**

**Response:**
We thank the reviewer for this important suggestion. The Methods section has been expanded to improve transparency and reproducibility of the modeling pipeline.

Specifically, we clarified that candidate predictors were prespecified based on clinical relevance, published literature, and guideline-supported risk factors, and that no post-hoc outcome-driven variable selection was performed to avoid information leakage. We further expanded the description of missing data handling using multiple imputation by chained equations (MICE), detailed the use of outcome-specific weighting to address class imbalance, and provided additional information regarding hyperparameter optimization using randomized search within five-fold cross-validation. The explainability framework was also clarified through the addition of a dedicated SHAP-based model interpretation section.

These revisions are reported in the Methods sections “Feature Selection and Data Preprocessing,” “Model Development and Validation,” and “Model Explainability and Clinical Interpretability.”

**Comment 2: Outcome definitions unclear (ischemic and bleeding definitions consistency)**

**Response:**

We thank the reviewer for this important clarification request.

The outcome definitions have been explicitly clarified in the revised Methods section under section of Study population and outcome. Composite ischemic events are now defined as myocardial infarction, ischemic stroke, stent thrombosis, and cardiovascular death. Major bleeding outcomes are defined according to Bleeding Academic Research Consortium (BARC) type 3 and type 5 criteria.

We additionally clarified that identical outcome definitions were applied across both the UAE development cohort and the MIMIC-IV external validation cohort using harmonized diagnostic and clinical coding mappings. Further details regarding ICD-based phenotyping algorithms used for outcome ascertainment are provided in Supplementary Table S1.

To reinforce consistency for readers, the same outcome definitions are also reiterated in the Results section under “Clinical Outcomes and Event Rates.”

**Comment 3: External validation comparability (MIMIC-IV ICU population differences)**

**Response:**
We thank the reviewer for this insightful comment. We have expanded the Methods, Results, Discussion, and Limitations sections to clarify differences between the UAE development cohort and the MIMIC-IV external validation cohort.

Revisions include:

• Explicit description of MIMIC-IV as a critical care (ICU-enriched) population in the Methods section.

• Additional reporting of baseline differences between cohorts, including age distribution, comorbidity burden, severity of illness, and event rates.

• Clarification that model performance was evaluated without recalibration to assess transportability across heterogeneous healthcare environments.

• Expanded Discussion and Limitations sections acknowledging that MIMIC-IV may not fully represent routine PCI populations and that external validation was intended to assess transportability rather than equivalence between cohorts.

These revisions provide additional context regarding cohort heterogeneity and support interpretation of the external validation findings.

**Comment 4: Calibration analysis insufficient**

**Response:**
Comment 4: Calibration analysis insufficient

We thank the reviewer for this important methodological suggestion. The evaluation of model performance has been expanded beyond discrimination metrics to include formal calibration assessment.

Revisions include:

• Addition of calibration intercept and calibration slope analyses.

• Inclusion of calibration plots for external validation with bootstrap-based uncertainty estimation.

• Expanded description of calibration assessment in the Methods section.

• Reporting of calibration performance in the Results section, demonstrating good agreement between predicted and observed event probabilities.

• Clarification that both discrimination and calibration were evaluated when assessing overall model performance. Calibration was assessed using calibration plots, calibration intercepts, and calibration slopes

These additions provide a more comprehensive assessment of predictive performance in accordance with contemporary recommendations for clinical prediction models.

## Comment 5: Economic evaluation insufficiently described

**Response:**
Comment 5: Economic evaluation insufficiently described

We thank the reviewer for this important comment. The Economic Evaluation section has been substantially expanded to improve transparency regarding the structure, assumptions, and interpretation of the economic analyses.

Revisions made:

• Clarified the use of a simplified decision-analytic framework comparing hypothetical AI-guided and guideline-based DAPT strategies.

• Added the analytic perspective (healthcare payer perspective) and time horizon (approximately 37 months corresponding to median follow-up).

• Detailed cost components, including antiplatelet therapy, management of major bleeding events, and hospitalization costs associated with ischemic events.

• Added the approach used to estimate quality-adjusted life-years (QALYs).

• Clarified that incremental cost-effectiveness ratios (ICERs) represent exploratory modeling estimates rather than definitive economic conclusions.

• Added deterministic one-way and probabilistic sensitivity analyses to evaluate parameter uncertainty.

• Explicitly acknowledged throughout the Methods, Results, and Discussion sections that economic estimates were derived from model-predicted event projections rather than observed treatment effects and therefore should be interpreted cautiously pending prospective validation.

**Comment 6: Clinical implementation unclear**

**Response:**

We appreciate this valuable suggestion. A dedicated paragraph has been added to the Discussion section to clarify potential implementation pathways and practical considerations for future clinical use.

Revisions made:

• Clarified that all predictor variables are routinely available from electronic health records shortly after PCI and therefore would not require specialized testing for implementation.

• Added discussion of integration within existing electronic health record systems to facilitate automated risk estimation at clinically relevant post-PCI time points.

• Clarified how individualized ischemic and bleeding risk predictions could support DAPT duration decision-making alongside existing guideline recommendations.

• Emphasized that the model is intended to function as a clinical decision-support tool that complements, rather than replaces, clinician judgment.

• Added discussion of implementation challenges, including workflow integration, clinician adoption, usability, governance considerations, and the need for prospective implementation studies before routine clinical deployment.

## Comment 7: Suggested literature citations

**Response:**
We thank the reviewer for these valuable suggestions. The recommended contemporary references (PMID: 39415380, PMID: 39054275, and PMID: 40467090) have been incorporated into the revised Discussion section. Specifically, we expanded the discussion of evolving post-PCI antithrombotic management strategies, including recent evidence supporting individualized treatment approaches, P2Y12 inhibitor monotherapy, and treatment de-escalation pathways. These additions provide broader clinical context for the proposed AI-based risk stratification framework and better position the present study within the current landscape of personalized antithrombotic therapy. The corresponding references have been added as References 32–34 in the revised manuscript.

**Comment 8: Conclusions too strong**

**Response:**
We agree with the reviewer and have revised the Conclusions section.

**Revision made:**

- Tonality adjusted to emphasize exploratory nature
- Clarified that economic and simulation results are not causal
- Strengthened cautious interpretation of AI-guided strategies

**RESPONSE TO REVIEWER #2** **Highlight in** 🟨

**Comment 1: Compare model to logistic regression**

**Response:**
We thank the reviewer for this important suggestion. Logistic regression was added as a benchmark model using the same predictor set as all machine learning approaches.

**Revisions made:**

- Logistic regression included as baseline comparator
- Results added to Results section, Table 4, Figure 2, and Supplementary Figure S4
- Clarified that logistic regression showed lower discrimination compared with weighted LightGBM
- Emphasized nonlinear modeling advantage

**Comment 2: Standard ROC curve required / clarify Figure 2**

**Response:**
We thank the reviewer for this suggestion. Figure 2 has been revised to include a standard ROC curve presentation.

**Revisions made:**

- Standard ROC curves added for all models
- Figure legend clarified
- Improved comparability across models

**Comment 3: Replace Figure 5 with forest plot**

**Response:**
We thank the reviewer for this suggestion. Figure 5 has been replaced with a standard forest plot.

**Revision made:**

- Effect estimates displayed using standard forest plot format
- Confidence intervals added
- Improved interpretability and reviewer compliance

**Comment 4:** Consider measuring the probability that clinicians give for the outcome in another study.

**Response:**
We thank the reviewer for this insightful suggestion. Clinician-estimated probabilities were not available within the retrospective datasets used in this study; therefore, direct comparison between model predictions and physician risk assessment could not be performed. We have added this as a study limitation and highlighted it as an important area for future research. The revised Discussion and Future Directions sections now recommend prospective studies comparing AI-generated risk estimates with clinician-assigned probabilities to evaluate the incremental value of AI-assisted decision support in post-PCI management.
