# Supplementary material for: A machine learning model for predicting ischemic and bleeding risk after percutaneous coronary intervention: Development and external validation
Source: PLoS One. 2026 Sep 15;21(9):e0353066. doi: 10.1371/journal.pone.0353066 (PMC13577505; doi:10.1371/journal.pone.0353066)
Supplement: Supplementary file 10 [file pone.0353066.s014.docx]

**Rebuttal Letter (Point-by-Point Response)**

**Header Example:**

**Rebuttal Letter for Manuscript ID [Your PLOS ONE ID]**
Title: *Artificial Intelligence-Guided Personalization of Dual Antiplatelet Therapy After Percutaneous Coronary Intervention: Development, External Validation, and Cost-Effectiveness Analysis*
Corresponding Author: Hassa Iftikhar

**Instructions:** For each point raised by the previous reviewers/editor, show what I changed, where, and provide a concise explanation. Use color coding or bold to match your manuscript highlighting (yellow/green/blue/purple).

| **Reviewer** | **Original Comment** | **Our Response** | **Manuscript Location / Action** |
| --- | --- | --- | --- |
| **R1 (Yellow)** | Clarify that projected clinical outcomes are simulations, not observed treatment effects. | Added a sentence in Methods under “Clinical Utility Assessment”: “The clinical outcomes represent scenario-based simulations derived from predicted risk distributions rather than observed treatment effects. No causal inference framework was applied, and the results should therefore be interpreted as exploratory modeling outputs rather than evidence of treatment benefit.” | Methods, Clinical Utility Assessment; also repeated in Discussion |
| **R1 (Yellow)** | Include clear limitations about retrospective design, ICU dataset, administrative codes, and simulated economic outcomes. | Added a dedicated Limitations paragraph in Discussion specifying these four points. | Discussion - Limitations Section |
| **R2 (Blue)** | Avoid overclaiming AI effectiveness; remove language implying causal benefits. | Reworded all AI claims in Introduction and Discussion to: “may support risk-guided strategies,” “improves risk prediction,” “hypothesis-generating,” and “requires prospective validation.” | Introduction & Discussion - multiple paragraphs |
| **R2 (Blue)** | Clarify that model is a risk prediction tool, not clinical decision-making system. | Added sentences: “However, the present model should be interpreted as a risk prediction tool rather than a clinical decision-making system.” and “If validated in prospective studies, clinical translation of such models would likely require integration into existing healthcare workflows.” | Discussion - Paragraphs 10-12 |
| **R3 (Green)** | Specify missing data handling and imputation. | Added MICE section: “Missing data were handled using multiple imputation by chained equations (MICE). Variables with >30% missingness were excluded. Ten imputed datasets were combined using Rubin’s rules.” | Methods - Feature Selection and Data Preprocessing; Supplementary Table S2 |
| **R3 (Green)** | Add hyperparameter tuning details for LightGBM. | Added: “Hyperparameter tuning for the LightGBM model was performed using five-fold cross-validation within the training dataset. Randomized search optimized learning rate, number of estimators, max tree depth, and feature fraction.” | Methods - Model Development and Validation |
| **R4 (Purple)** | Outcome phenotyping unclear; ICD codes missing. | Added explicit ICD-9/ICD-10 mappings and reference to Supplementary Table S1. | Methods - Study Population & Outcome; Supplementary Table S1 |
| **R4 (Purple)** | External validation credibility and reproducibility concerns. | Clarified MIMIC-IV access, coding, and harmonization; highlighted limitations due to ICU-specific population; added sensitivity analyses using alternative definitions. | Methods - Study Population & Outcome; Discussion - Limitations |

**Note:** All changes are highlighted in manuscript using the yellow/blue/green/purple color code as discussed.
