# Supplementary material for: A machine learning model for predicting ischemic and bleeding risk after percutaneous coronary intervention: Development and external validation
Source: PLoS One. 2026 Sep 15;21(9):e0353066. doi: 10.1371/journal.pone.0353066 (PMC13577505; doi:10.1371/journal.pone.0353066)
Supplement: Peer review history

# Author comment: Author response to Decision Letter 0

**DOI:** 10.1371/journal.pone.0353066.r001

**Transfer Alert**

This paper was transferred from another journal. As a result, its full editorial history (including decision letters, peer reviews and author responses) may not be present.

12 Mar 2026

Attachment Submitted filename: Rebuttal Letter.docx

# Peer review: Decision Letter 0

**DOI:** 10.1371/journal.pone.0353066.r002

**Authors:** Chiara Lazzeri

4 May 2026

Dear Dr. Iftikhar,

Please submit your revised manuscript by Jun 17 2026 11:59PM. If you will need more time than this to complete your revisions, please reply to this message or contact the journal office at plosone@plos.org. When you're ready to submit your revision, log on to https://www.editorialmanager.com/pone/ and select the 'Submissions Needing Revision' folder to locate your manuscript file.

- A letter that responds to each point raised by the academic editor and reviewer(s). You should upload this letter as a separate file labeled 'Response to Reviewers'.
- A marked-up copy of your manuscript that highlights changes made to the original version. You should upload this as a separate file labeled 'Revised Manuscript with Track Changes'.
- An unmarked version of your revised paper without tracked changes. You should upload this as a separate file labeled 'Manuscript'.

If you would like to make changes to your financial disclosure, please include your updated statement in your cover letter. Guidelines for resubmitting your figure files are available below the reviewer comments at the end of this letter.

If applicable, we recommend that you deposit your laboratory protocols in protocols.io to enhance the reproducibility of your results. Protocols.io assigns your protocol its own identifier (DOI) so that it can be cited independently in the future. For instructions see: https://journals.plos.org/plosone/s/submission-guidelines#loc-laboratory-protocols. Additionally, PLOS ONE offers an option for publishing peer-reviewed Lab Protocol articles, which describe protocols hosted on protocols.io. Read more information on sharing protocols at https://plos.org/protocols?utm_medium=editorial-email&utm_source=authorletters&utm_campaign=protocols.

As the corresponding author, your ORCID iD is verified in the submission system and will appear in the published article. PLOS supports the use of ORCID, and we encourage all coauthors to register for an ORCID iD and use it as well. Please encourage your coauthors to verify their ORCID iD within the submission system before final acceptance, as unverified ORCID iDs will not appear in the published article. *Only*  the individual author can complete the verification step; PLOS staff *cannot*  verify ORCID iDs on behalf of authors.

We look forward to receiving your revised manuscript.

Kind regards,

Chiara Lazzeri

Academic Editor

PLOS One

Journal Requirements:

When submitting your revision, we need you to address these additional requirements.

1. Please ensure that your manuscript meets PLOS ONE's style requirements, including those for file naming. The PLOS ONE style templates can be found at

https://journals.plos.org/plosone/s/file?id=wjVg/PLOSOne_formatting_sample_main_body.pdf and

https://journals.plos.org/plosone/s/file?id=ba62/PLOSOne_formatting_sample_title_authors_affiliations.pdf

2. Please note that PLOS One has specific guidelines on code sharing for submissions in which author-generated code underpins the findings in the manuscript. In these cases, all author-generated code must be made available without restrictions upon publication of the work. Please review our guidelines at https://journals.plos.org/plosone/s/materials-and-software-sharing#loc-sharing-code and ensure that your code is shared in a way that follows best practice and facilitates reproducibility and reuse.

3. In the online submission form, you indicated that “ Additional data used for model development and external validation were obtained from third-party sources and are subject to access restrictions. Specifically, access to the MIMIC-IV database is governed by the PhysioNet Credentialed Health Data Access framework due to the inclusion of sensitive health information, and researchers must obtain appropriate authorization to access these data. MIMIC-IV data are publicly available to qualified researchers through credentialed access via PhysioNet (https://physionet.org/), subject to completion of required data use training and approval under the PhysioNet Credentialed Health Data Access program. Data derived from regional healthcare repositories were used in accordance with their respective data-sharing policies. The UAE clinical dataset used for external validation contains patient-level health information subject to institutional privacy and regulatory restrictions and therefore cannot be publicly shared. Detailed descriptions of cohort derivation, variable definitions, preprocessing steps, and analytical methods are provided in the Supplementary Materials to facilitate transparency and reproducibility. Aggregated summary statistics and model development code used for analysis will be made available by the corresponding author upon reasonable request, subject to institutional and ethical approval. Requests for access to aggregated data or analytic code should be directed to the corresponding author.”

All PLOS journals now require all data underlying the findings described in their manuscript to be freely available to other researchers, either 1. In a public repository, 2. Within the manuscript itself, or 3. Uploaded as supplementary information.

This policy applies to all data except where public deposition would breach compliance with the protocol approved by your research ethics board. If your data cannot be made publicly available for ethical or legal reasons (e.g., public availability would compromise patient privacy), please explain your reasons on resubmission and your exemption request will be escalated for approval.

4. When completing the data availability statement of the submission form, you indicated that you will make your data available on acceptance. We strongly recommend all authors decide on a data sharing plan before acceptance, as the process can be lengthy and hold up publication timelines. Please note that, though access restrictions are acceptable now, your entire data will need to be made freely accessible if your manuscript is accepted for publication. This policy applies to all data except where public deposition would breach compliance with the protocol approved by your research ethics board. If you are unable to adhere to our open data policy, please kindly revise your statement to explain your reasoning and we will seek the editor's input on an exemption. Please be assured that, once you have provided your new statement, the assessment of your exemption will not hold up the peer review process.

5. We note that you have indicated that there are restrictions to data sharing for this study. For studies involving human research participant data or other sensitive data, we encourage authors to share de-identified or anonymized data. However, when data cannot be publicly shared for ethical reasons, we allow authors to make their data sets available upon request. For information on unacceptable data access restrictions, please see http://journals.plos.org/plosone/s/data-availability#loc-unacceptable-data-access-restrictions.

Before we proceed with your manuscript, please address the following prompts:

a) If there are ethical or legal restrictions on sharing a de-identified data set, please explain them in detail (e.g., data contain potentially identifying or sensitive patient information, data are owned by a third-party organization, etc.) and who has imposed them (e.g., a Research Ethics Committee or Institutional Review Board, etc.). Please also provide contact information for a data access committee, ethics committee, or other institutional body to which data requests may be sent.

b) If there are no restrictions, please upload the minimal anonymized data set necessary to replicate your study findings to a stable, public repository and provide us with the relevant URLs, DOIs, or accession numbers. Please see http://www.bmj.com/content/340/bmj.c181.long for guidelines on how to de-identify and prepare clinical data for publication. For a list of recommended repositories, please see https://journals.plos.org/plosone/s/recommended-repositories. You also have the option of uploading the data as Supporting Information files, but we would recommend depositing data directly to a data repository if possible.

Please update your Data Availability statement in the submission form accordingly.

6. Please amend either the title on the online submission form (via Edit Submission) or the title in the manuscript so that they are identical.

7. Please amend either the abstract on the online submission form (via Edit Submission) or the abstract in the manuscript so that they are identical.

8. Please include a separate caption for each figure in your manuscript.

9. Please include your tables as part of your main manuscript and remove the individual files. Please note that supplementary tables (should remain/ be uploaded) as separate "supporting information" files

10. We notice that your supplementary figures are uploaded with the file type 'Other'. Please amend the file type to 'Supporting Information'. Please ensure that each Supporting Information file has a legend listed in the manuscript after the references list.

11. If the reviewer comments include a recommendation to cite specific previously published works, please review and evaluate these publications to determine whether they are relevant and should be cited. There is no requirement to cite these works unless the editor has indicated otherwise.

[Note: HTML markup is below. Please do not edit.]

Reviewers' comments:

Reviewer's Responses to Questions

**Comments to the Author**

1. Is the manuscript technically sound, and do the data support the conclusions?

Reviewer #1: Yes

Reviewer #2: Yes

**********

2. Has the statistical analysis been performed appropriately and rigorously?

Reviewer #1: Yes

Reviewer #2: Yes

**********

3. Have the authors made all data underlying the findings in their manuscript fully available?

The PLOS Data policy

Reviewer #1: Yes

Reviewer #2: Yes

**********

4. Is the manuscript presented in an intelligible fashion and written in standard English?

Reviewer #1: Yes

Reviewer #2: Yes

**********

Reviewer #1: This manuscript presents the development and external validation of an AI model for predicting ischemic and bleeding risk after PCI, together with a cost-effectiveness analysis comparing AI-guided versus conventional DAPT strategies. The topic is timely and clinically relevant, and the effort to combine risk prediction with economic evaluation is commendable. I have the following comments for the authors.

Although the use of a LightGBM-based approach is appropriate, the description of the model development process remains insufficient. It is not entirely clear how variables were selected, whether this process was prespecified or data-driven, and how missing data were handled. Similarly, the strategy for hyperparameter tuning is only briefly mentioned, and the approach to class imbalance, although referenced, is not fully detailed. The manuscript repeatedly refers to “explainable AI,” but the methods used to achieve explainability are not described in sufficient depth. A more detailed description of the modeling pipeline is needed, ideally in line with TRIPOD-AI recommendations.

The manuscript refers to composite ischemic events and major bleeding, but these outcomes are not defined with sufficient precision. It remains unclear whether ischemic events include myocardial infarction, stroke, or stent thrombosis, and whether bleeding is classified according to BARC, TIMI, or another system. Moreover, it is not evident whether outcome definitions are consistent between the derivation and validation cohorts. This aspect requires clarification.

While the use of the MIMIC-IV database is innovative, this dataset represents an intensive care population and may differ significantly from a typical PCI cohort. Potential differences in patient characteristics, and outcome definitions may limit the interpretation of this validation as a true external validation. A more detailed comparison between the derivation and validation cohorts would help the reader understand the degree of comparability and the extent to which the results reflect generalizability across heterogeneous datasets.

With regard to model performance, the manuscript primarily focuses on discrimination metrics, reporting AUROC values that appear favorable. However, the evaluation remains incomplete without a more thorough assessment of calibration. Key metrics such as calibration slope, intercept, or Brier score are not reported. In addition, although decision curve analysis is included, its clinical interpretation remains limited. A more comprehensive evaluation of model performance would strengthen the credibility of the findings.

The cost-effectiveness analysis is an interesting addition but has limitations. The structure of the economic model is not described in sufficient detail, and key assumptions, time horizons, and cost inputs are not fully transparent. Most importantly, the economic conclusions are based on predicted rather than observed events, which introduces a substantial degree of uncertainty. This aspect should be more explicitly acknowledged, and the economic analysis should be described and interpreted with greater caution.

While the model demonstrates promising performance, the manuscript does not clearly explain how it could be implemented in clinical practice. It is not evident whether all required variables are readily available at the point of care, nor how the model would concretely influence therapeutic decision-making compared with current guideline-based strategies. A more explicit discussion of potential implementation pathways and barriers would enhance the clinical relevance of the work.

I would suggest citing a recent expert review article (PMID: 39415380), which is highly relevant to the topic and would provide readers with a broader and up-to-date overview of risk stratification tools and antithrombotic decision-making after PCI.

Along the same lines, the manuscript would benefit from a more comprehensive integration of recent literature on antithrombotic therapy after PCI. In particular, the authors should discuss recent contributions on P2Y12 inhibitor monotherapy early after PCI (PMID: 39054275) and as long-term secondary prevention strategy in patients with CAD (PMID: 39054275, PMID: 40467090) to better position the present work within the evolving landscape of personalized antithrombotic therapy.

The conclusions appear somewhat overstated in light of the study design. Given the absence of real-world outcome data and the exploratory nature of the economic analysis, the conclusions should be more cautious.

Reviewer #2: Please compare your model to a logistic regression model.

Please provide a standard ROC curve in place of Figure 2 or at least explain Figure 2.

Please replace Figure 5 with a standard forest plot.

Consider measuring the probability that clinicians give for the outcome in another study.

**********

what does this mean?). If published, this will include your full peer review and any attached files.

If you choose “no”, your identity will remain anonymous but your review may still be made public.

**Do you want your identity to be public for this peer review?**  For information about this choice, including consent withdrawal, please see our Privacy Policy

Reviewer #1: No

Reviewer #2: No

**********

[NOTE: If reviewer comments were submitted as an attachment file, they will be attached to this email and accessible via the submission site. Please log into your account, locate the manuscript record, and check for the action link "View Attachments". If this link does not appear, there are no attachment files.]

To ensure your figures meet our technical requirements, please review our figure guidelines: https://journals.plos.org/plosone/s/figures

You may also use PLOS’s free figure tool, NAAS, to help you prepare publication quality figures: https://journals.plos.org/plosone/s/figures#loc-tools-for-figure-preparation.

NAAS will assess whether your figures meet our technical requirements by comparing each figure against our figure specifications.

# Author comment: Author response to Decision Letter 1

**DOI:** 10.1371/journal.pone.0353066.r003

6 Jun 2026

RESPONSE TO REVIEWER #1 Highlight in 🟢

Comment 1: Insufficient model development detail (variables, missing data, hyperparameter tuning, explainability)

Response:

We thank the reviewer for this important suggestion. The Methods section has been expanded to improve transparency and reproducibility of the modeling pipeline.

Specifically, we clarified that candidate predictors were prespecified based on clinical relevance, published literature, and guideline-supported risk factors, and that no post-hoc outcome-driven variable selection was performed to avoid information leakage. We further expanded the description of missing data handling using multiple imputation by chained equations (MICE), detailed the use of outcome-specific weighting to address class imbalance, and provided additional information regarding hyperparameter optimization using randomized search within five-fold cross-validation. The explainability framework was also clarified through the addition of a dedicated SHAP-based model interpretation section.

These revisions are reported in the Methods sections “Feature Selection and Data Preprocessing,” “Model Development and Validation,” and “Model Explainability and Clinical Interpretability.”

Comment 2: Outcome definitions unclear (ischemic and bleeding definitions consistency)

Response:

We thank the reviewer for this important clarification request.

The outcome definitions have been explicitly clarified in the revised Methods section under section of Study population and outcome. Composite ischemic events are now defined as myocardial infarction, ischemic stroke, stent thrombosis, and cardiovascular death. Major bleeding outcomes are defined according to Bleeding Academic Research Consortium (BARC) type 3 and type 5 criteria.

We additionally clarified that identical outcome definitions were applied across both the UAE development cohort and the MIMIC-IV external validation cohort using harmonized diagnostic and clinical coding mappings. Further details regarding ICD-based phenotyping algorithms used for outcome ascertainment are provided in Supplementary Table S1.

To reinforce consistency for readers, the same outcome definitions are also reiterated in the Results section under “Clinical Outcomes and Event Rates.”

Comment 3: External validation comparability (MIMIC-IV ICU population differences)

Response:

We thank the reviewer for this insightful comment. We have expanded the Methods, Results, Discussion, and Limitations sections to clarify differences between the UAE development cohort and the MIMIC-IV external validation cohort.

Revisions include:

• Explicit description of MIMIC-IV as a critical care (ICU-enriched) population in the Methods section.

• Additional reporting of baseline differences between cohorts, including age distribution, comorbidity burden, severity of illness, and event rates.

• Clarification that model performance was evaluated without recalibration to assess transportability across heterogeneous healthcare environments.

• Expanded Discussion and Limitations sections acknowledging that MIMIC-IV may not fully represent routine PCI populations and that external validation was intended to assess transportability rather than equivalence between cohorts.

These revisions provide additional context regarding cohort heterogeneity and support interpretation of the external validation findings.

Comment 4: Calibration analysis insufficient

Response:

Comment 4: Calibration analysis insufficient

We thank the reviewer for this important methodological suggestion. The evaluation of model performance has been expanded beyond discrimination metrics to include formal calibration assessment.

Revisions include:

• Addition of calibration intercept and calibration slope analyses.

• Inclusion of calibration plots for external validation with bootstrap-based uncertainty estimation.

• Expanded description of calibration assessment in the Methods section.

• Reporting of calibration performance in the Results section, demonstrating good agreement between predicted and observed event probabilities.

• Clarification that both discrimination and calibration were evaluated when assessing overall model performance. Calibration was assessed using calibration plots, calibration intercepts, and calibration slopes

These additions provide a more comprehensive assessment of predictive performance in accordance with contemporary recommendations for clinical prediction models.

Comment 5: Economic evaluation insufficiently described

Response:

Comment 5: Economic evaluation insufficiently described

We thank the reviewer for this important comment. The Economic Evaluation section has been substantially expanded to improve transparency regarding the structure, assumptions, and interpretation of the economic analyses.

Revisions made:

• Clarified the use of a simplified decision-analytic framework comparing hypothetical AI-guided and guideline-based DAPT strategies.

• Added the analytic perspective (healthcare payer perspective) and time horizon (approximately 37 months corresponding to median follow-up).

• Detailed cost components, including antiplatelet therapy, management of major bleeding events, and hospitalization costs associated with ischemic events.

• Added the approach used to estimate quality-adjusted life-years (QALYs).

• Clarified that incremental cost-effectiveness ratios (ICERs) represent exploratory modeling estimates rather than definitive economic conclusions.

• Added deterministic one-way and probabilistic sensitivity analyses to evaluate parameter uncertainty.

• Explicitly acknowledged throughout the Methods, Results, and Discussion sections that economic estimates were derived from model-predicted event projections rather than observed treatment effects and therefore should be interpreted cautiously pending prospective validation.

Comment 6: Clinical implementation unclear

Response:

We appreciate this valuable suggestion. A dedicated paragraph has been added to the Discussion section to clarify potential implementation pathways and practical considerations for future clinical use.

Revisions made:

• Clarified that all predictor variables are routinely available from electronic health records shortly after PCI and therefore would not require specialized testing for implementation.

• Added discussion of integration within existing electronic health record systems to facilitate automated risk estimation at clinically relevant post-PCI time points.

• Clarified how individualized ischemic and bleeding risk predictions could support DAPT duration decision-making alongside existing guideline recommendations.

• Emphasized that the model is intended to function as a clinical decision-support tool that complements, rather than replaces, clinician judgment.

• Added discussion of implementation challenges, including workflow integration, clinician adoption, usability, governance considerations, and the need for prospective implementation studies before routine clinical deployment.

Comment 7: Suggested literature citations

Response:

We thank the reviewer for these valuable suggestions. The recommended contemporary references (PMID: 39415380, PMID: 39054275, and PMID: 40467090) have been incorporated into the revised Discussion section. Specifically, we expanded the discussion of evolving post-PCI antithrombotic management strategies, including recent evidence supporting individualized treatment approaches, P2Y12 inhibitor monotherapy, and treatment de-escalation pathways. These additions provide broader clinical context for the proposed AI-based risk stratification framework and better position the present study within the current landscape of personalized antithrombotic therapy. The corresponding references have been added as References 32–34 in the revised manuscript.

Comment 8: Conclusions too strong

Response:

We agree with the reviewer and have revised the Conclusions section.

Revision made:

• Tonality adjusted to emphasize exploratory nature

• Clarified that economic and simulation results are not causal

• Strengthened cautious interpretation of AI-guided strategies

RESPONSE TO REVIEWER #2 Highlight in 🟨

Comment 1: Compare model to logistic regression

Response:

We thank the reviewer for this important suggestion. Logistic regression was added as a benchmark model using the same predictor set as all machine learning approaches.

Revisions made:

• Logistic regression included as baseline comparator

• Results added to Results section, Table 4, Figure 2, and Supplementary Figure S4

• Clarified that logistic regression showed lower discrimination compared with weighted LightGBM

• Emphasized nonlinear modeling advantage

Comment 2: Standard ROC curve required / clarify Figure 2

Response:

We thank the reviewer for this suggestion. Figure 2 has been revised to include a standard ROC curve presentation.

Revisions made:

• Standard ROC curves added for all models

• Figure legend clarified

• Improved comparability across models

Comment 3: Replace Figure 5 with forest plot

Response:

We thank the reviewer for this suggestion. Figure 5 has been replaced with a standard forest plot.

Revision made:

• Effect estimates displayed using standard forest plot format

• Confidence intervals added

• Improved interpretability and reviewer compliance

Comment 4: Consider measuring the probability that clinicians give for the outcome in another study.

Response:

We thank the reviewer for this insightful suggestion. Clinician-estimated probabilities were not available within the retrospective datasets used in this study; therefore, direct comparison between model predictions and physician risk assessment could not be performed. We have added this as a study limitation and highlighted it as an important area for future research. The revised Discussion and Future Directions sections now recommend prospective studies comparing AI-generated risk estimates with clinician-assigned probabilities to evaluate the incremental value of AI-assisted decision support in post-PCI management.

Attachment Submitted filename: Response Letter.docx

# Editor report: Decision Letter 1

**DOI:** 10.1371/journal.pone.0353066.r004

**Authors:** Chiara Lazzeri

18 Jun 2026

A Machine Learning Model for Predicting Ischemic and Bleeding Risk After Percutaneous Coronary Intervention: Development and External Validation

PONE-D-26-12102R1

Dear Dr. Iftikhar,

We’re pleased to inform you that your manuscript has been judged scientifically suitable for publication and will be formally accepted for publication once it meets all outstanding technical requirements.

Within one week, you’ll receive an e-mail detailing the required amendments. When these have been addressed, you’ll receive a formal acceptance letter and your manuscript will be scheduled for publication.

An invoice will be generated when your article is formally accepted. Please note, if your institution has a publishing partnership with PLOS and your article meets the relevant criteria, all or part of your publication costs will be covered. Please make sure your user information is up-to-date by logging into Editorial Manager at Editorial Manager® and clicking the ‘Update My Information' link at the top of the page. For questions related to billing, please contact billing support.

If your institution or institutions have a press office, please notify them about your upcoming paper to help maximize its impact. If they’ll be preparing press materials, please inform our press team as soon as possible -- no later than 48 hours after receiving the formal acceptance. Your manuscript will remain under strict press embargo until 2 pm Eastern Time on the date of publication. For more information, please contact onepress@plos.org.

Kind regards,

Chiara Lazzeri

Academic Editor

PLOS One

Additional Editor Comments (optional):

Reviewers' comments:

# Editor report: Acceptance letter

**DOI:** 10.1371/journal.pone.0353066.r005

**Authors:** Chiara Lazzeri

PONE-D-26-12102R1

PLOS One

Dear Dr. Iftikhar,

I'm pleased to inform you that your manuscript has been deemed suitable for publication in PLOS One. Congratulations! Your manuscript is now being handed over to our production team.

At this stage, our production department will prepare your paper for publication. This includes ensuring the following:

* All references, tables, and figures are properly cited

* All relevant supporting information is included in the manuscript submission,

* There are no issues that prevent the paper from being properly typeset

You will receive further instructions from the production team, including instructions on how to review your proof when it is ready. Please keep in mind that we are working through a large volume of accepted articles, so please give us a few days to review your paper and let you know the next and final steps.

Lastly, if your institution or institutions have a press office, please let them know about your upcoming paper now to help maximize its impact. If they'll be preparing press materials, please inform our press team within the next 48 hours. Your manuscript will remain under strict press embargo until 2 pm Eastern Time on the date of publication. For more information, please contact onepress@plos.org.

You will receive an invoice from PLOS for your publication fee after your manuscript has reached the completed accept phase. If you receive an email requesting payment before acceptance or for any other service, this may be a phishing scheme. Learn how to identify phishing emails and protect your accounts at https://explore.plos.org/phishing.

If we can help with anything else, please email us at customercare@plos.org.

Thank you for submitting your work to PLOS One and supporting open access.

Kind regards,

PLOS One Editorial Office Staff

on behalf of

Dr. Chiara Lazzeri

Academic Editor

PLOS One
